# Pharmacogenomics of antibiotic-induced hypersensitivity reactions: current evidence and implications in clinical practice

**DOI:** 10.3389/fphar.2025.1651909

**Published:** 2025-10-01

**Authors:** Mohitosh Biswas, Murshadul Alam Murad, Md. Ismail Hossain Ashik, Maliheh Ershadian, Chonlaphat Sukasem

**Affiliations:** ^1^ Department of Pharmacy, Faculty of Science, University of Rajshahi, Rajshahi, Bangladesh; ^2^ Division of Pharmacogenomics and Personalized Medicine, Department of Pathology, Faculty of Medicine Ramathibodi Hospital, Mahidol University, Bangkok, Thailand; ^3^ Laboratory for Pharmacogenomics, Somdech Phra Debaratana Medical Center (SDMC), Ramathibodi Hospital, Bangkok, Thailand; ^4^ Pharmacogenomics and Precision Medicine, The Preventive Genomics and Family Check-Up Services Center, Bumrungrad International Hospital, Bangkok, Thailand; ^5^ Faculty of Pharmaceutical Sciences, Burapha University, Saensuk, Chonburi, Thailand

**Keywords:** antibiotics, hypersensitivity, severe cutaneous adverse drug reactions, liver injury, pharmacogenomics, precision medicine

## Abstract

Adverse drug reactions (ADRs) are gradually becoming a concerning health threat worldwide in patients undergoing acute or chronic therapy. Antibiotics are the main drugs that cause immune-mediated ADRs, such as severe cutaneous adverse reactions (SCARs), allergic reactions, and organ-specific diseases, representing a significant threat to patient safety. In this review, we present the current genetic evidence available for antibiotic-related toxicities from a pharmacogenomics (PGx) perspective. We also explore the current state of PGx-based dosing recommendations and the factors limiting their widespread application in routine clinical practice. Through a systematic literature review, this study identified at least 12 antibiotic–gene pairs (amikacin–*MT-RNR1*, gentamicin–*MT-RNR1*, kanamycin–*MT-RNR1*, streptomycin–*MT-RNR1*, neomycin–*MT-RNR1*, tobramycin–*MT-RNR1*, isoniazid–*NAT2*, dapsone–*HLA-B*, co-trimoxazole–*HLA-B*, *HLA-C*, flucloxacillin–*HLA-B*, daunorubicin–*SLC28A3*, and doxorubicin–*SLC28A3*) with moderate to high Pharmacogenomics Knowledgebase (PharmGKB) evidence levels for toxicity. However, PGx-based dosing guidelines, as recommended by the Clinical Pharmacogenetics Implementation Consortium (CPIC), the Dutch Pharmacogenetics Working Group (DPWG), and the Canadian Pharmacogenomics Network for Drug Safety (CPNDS), are currently available only for the following antibiotic–gene pairs*:* amikacin, gentamicin, kanamycin, streptomycin*,* neomycin, and tobramycin–*MT-RNR1*; flucloxacillin–*HLA-B*; dapsone–*G6PD*; nitrofurantoin–*G6PD*; and daunorubicin and doxorubicin–*RARG*, *SLC28A3*, and *UGT1A6*. Despite the established and growing genetic evidence for toxicity, particularly for Co-trimoxazole-induced SCARs by *HLA-B* and *HLA-C*, dapsone-induced SCARs by the *HLA-B*, and isoniazid-induced liver injury by the *NAT2*, insufficient approaches are being undertaken to translate these findings into routine clinical practice. The lack of validation of preliminary genetic associations, due to the scarcity of proper follow-up and large-scale replication, remains a key setback for PGx-based implementation of antibiotic therapy in clinical settings. More focused clinical studies, cost-effectiveness analyses, and polygenic risk score development are required to enable the PGx-based clinical use of antibiotics and optimize both safety and effectiveness in achieving precision medicine.

## 1 Introduction

Adverse drug reactions (ADRs) are gradually becoming a concerning health threat worldwide in patients undergoing acute or chronic therapy (Osanlou et al., 2018). Rawlins and Thompson grouped ADRs into two types: dose-dependent and predictable reactions (type A) and unpredictable dose-independent reactions (type B) (Dekker et al., 1997). Hypersensitivity reaction, a type-B ADR, is produced by cellular mediators released through both immunological and non-immune mechanisms (Doña et al., 2012). Allergic reactions are hypersensitivity reactions involving either an immunoglobulin E (IgE)-mediated or non-IgE (e.g., T cell)-mediated mechanism (Johansson et al., 2004). Severe cutaneous adverse reactions (SCARs) are potentially fatal T-cell-mediated delayed allergic reactions (Peter et al., 2017). The most prevalent SCARs, contributing to over 85% of the SCARs occurring in adults, are drug reaction with eosinophilia and systemic symptoms (DRESS), Stevens–Johnson syndrome (SJS), toxic epidermal necrolysis (TEN), and acute generalized exanthematous pustulosis (AGEP) (Duong et al., 2017; Sassolas et al., 2010).

A high estimated mortality ranging from 10% to 40% for SJS/TEN, <5% for AGEP and 2%–10% for DRESS was reported (Chen et al., 2010; Duong et al., 2017; Firoz et al., 2012; Husain et al., 2013; Kloypan et al., 2021; Owen and Jones, 2021; Schneck et al., 2008). Globally, the prevalence of SCARs was said to be 0.4–1.2 per million/years (Verma et al., 2013). Nevertheless, a racial discrepancy in the prevalence of SCARs has also been recorded. For example, the incidence was reported to be as high as 1.53–1.89 per million/year in the German population, whereas among the Filipino population, the rate of SCARs was reported to be 6.25/10,000 people from 2011 to 2015 (Guzman and Paliza, 2018; Mockenhaupt, 2012; Tempark et al., 2022). Additionally, the prevalence of TEN and SJS was estimated to be 0.4–1.2 and 1-6 per million/year, respectively, among the European population, while the rate was 0.94–1.45 and 3.96–5.03 per million/year, respectively, for Koreans (Yang et al., 2016; Kang et al., 2021; Duong et al., 2017).

Antibiotics are the main drugs that cause immune-mediated ADRs, such as SCARs, allergic reactions, and organ-specific diseases, representing an indisputable threat to patient safety (Blumenthal et al., 2019). Several antibiotics (e.g., beta-lactams, co-trimoxazole, vancomycin, and dapsone) have been associated with drug-induced hypersensitivity reactions (DIHRs) and have been associated with different genetic variants (Konvinse et al., 2019; Sukasem et al., 2020; Tempark et al., 2017; Wang et al., 2024a). Apart from DIHRs, other ADRs are also attributable to antibiotics. For example, anti-tuberculosis drug-induced hepatotoxicity (ATDH) represents an important clinical challenge as it is associated with treatment failure and increased mortality. The risk of developing hepatotoxicity ranges from 2% to 18% (Devarbhavi et al., 2010; Ramappa and Aithal, 2013). Cardiotoxicity is another important ADR related to anthracycline antibiotics and is deemed the most critical ADR in childhood cancer therapy, contributing to substantial mortality and morbidity (Lipshultz et al., 2008). In addition to nephrotoxicity, cochleotoxicity (sensorineural hearing loss) and vestibulotoxicity are the well-established side effects of aminoglycosides, which are typically dose-dependent and occur in the long-term use of high-dose drugs. However, certain individuals have been reported to be sensitive to aminoglycoside-induced hearing loss, even with single doses, resulting in profound bilateral sensorineural hearing loss (Mcdermott et al., 2022; Dean and Kane, 2018).

Recently developed cutting-edge technologies have identified the molecular mechanisms of underlying DIHRs and other ADRs. Therefore, in this article, we present the current genetic evidence from a pharmacogenomics (PGx) perspective. We also explore how these PGx–antibiotic associations can be more effectively translated into clinical practice to optimize antibiotic safety or efficacy, thereby serving as a cornerstone of antibiotic precision medicine.

## 2 Methods

### 2.1 Literature searching

Following the PRISMA guidelines, an extensive literature search was undertaken on PubMed on 25/5/2025 with the following keywords: pharmacogenomics, hypersensitivity, antibiotics, beta-lactam, sulfonamide, co-trimoxazole, dapsone, vancomycin, fluoroquinolone, anticancer antibiotics, macrolide, aminoglycoside, cephalosporins, tetracyclines, and anti-tubercular drugs to identify relevant articles (Page et al., 2021). Articles were included if 1 the study was performed on human subjects, 2 the study assessed the pharmacogenomic association of an antibiotic drug, and 3 the study evaluated the association of any gene or variant with antibiotic-induced hypersensitivity or adverse reactions. Studies were excluded if 1 the genetic assessment was conducted only computationally, 2 the study reported the genetic frequency without associating the findings with any drug, 3 the analysis was *in vitro* or the studies was conducted in an animal model, and 4 the publication was something other than a research article (e.g., review articles, meta-analysis, book chapter, editorial, case report, letter, and conference paper),

We utilized Rayyan QCRI, a web-based tool for systematic reviews, to select the primary studies (Ouzzani et al., 2016). We obtained the full texts of initially selected studies and reviewed them carefully to determine the final set of studies for inclusion. Two researchers independently performed the study selection using Rayyan QCRI software, and any disagreements during data extraction were resolved through mutual discussion.

### 2.2 Identification of the PGx-based evidence level, drug label, and therapeutic and testing guidelines for antibiotics

To assess the current state of PGx-based evidence for gene variants involved in the toxicity, metabolism/pharmacokinetics (PK), and efficacy of antibiotics, we utilized clinical annotations provided by the Pharmacogenomics Knowledgebase (PharmGKB), which is a comprehensive PGx resource managed by Stanford University to support, expand, and promote the implementation and education of PGx knowledge. PGx-based drug label information for the antibiotics was sourced from various internationally acknowledged pharmacogenetics working bodies, namely, the Health Canada Santé Canada (HCSC)-approved drug label, the US Food and Drug Administration (FDA)-approved drug label, the Swissmedic (Swiss Agency of Therapeutic Products)-approved drug label, the Pharmaceuticals and Medical Devices Agency (Japan) (PMDA)-approved drug label, and the European Medicines Agency (EMA)-approved drug label. We accessed all the information from the PharmGKB website (Barbarino et al., 2018). To obtain current information on therapeutic and testing guidelines for antibiotics, we searched different guideline-providing PGx working groups and included recommendations from the Clinical Pharmacogenetics Implementation Consortium (CPIC), the Dutch Pharmacogenetics Working Group (DPWG), and the Canadian Pharmacogenomics Network for Drug Safety (CPNDS) (CPIC, 2025; CPNDS, 2025; DPWG, 2025a).

## 3 Results

### 3.1 Literature search results

**FIGURE 1:**
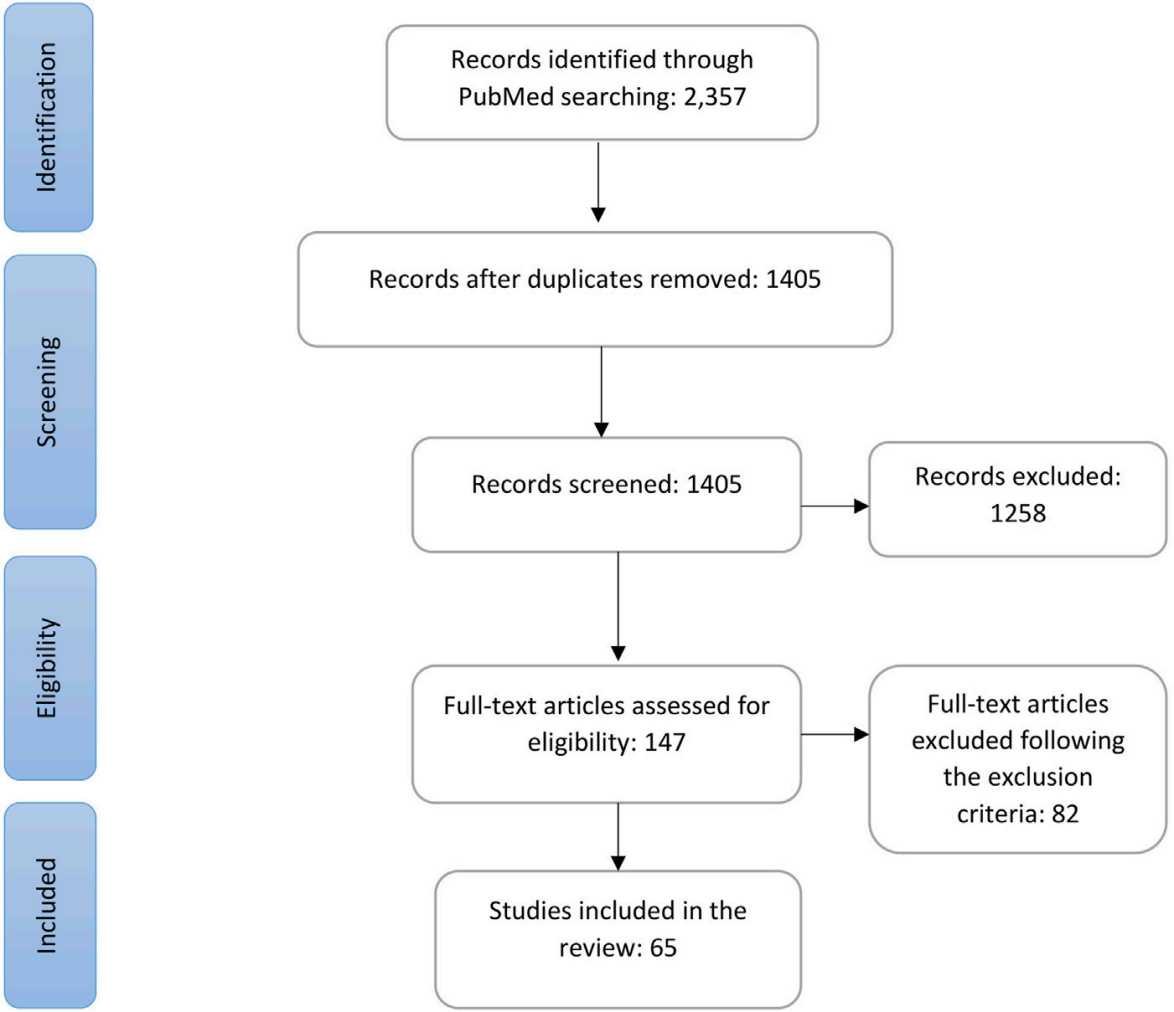
PRISMA flowchart of the selection of studies.

The strategic search using the aforementioned keywords generated 2,357 records, and after removal of duplicates, 1,405 remained for screening. Through initial screening with title and abstract, we excluded 1,258, and after another round of screening, we identified 147 articles for full-text eligibility assessment. Following the predefined inclusion and exclusion criteria (detailed in the Section 2), we identified 65 articles that examined the PGx associations of genes with the DIHRs and other adverse effects of antibiotics for inclusion in this review. The whole selection process is shown in a PRISMA flowchart in Figure 1.

Of the identified 65 articles, PGx assessments are presented for beta-lactams in 8 studies, anti-tuberculosis drugs in 25 studies, anticancer antibiotics in 13 studies, sulfonamides in 6 studies, aminoglycosides in 4 studies, and other antibiotics in the remaining 9 studies. Table 1 summarizes the key PGx associations for antibiotics from the included studies.

**TABLE 1:** Overview of the included studies that reported significant PGx associations of different genes/variants for antibiotic drugs.

Drug	Gene	OR (95% CI)	*p*-value	Adverse effect	Population/race	Reference
Beta-lactams						
Amoxicillin, benzyl penicillin, amoxicillin–clavulanic acid, and cephalosporins	*LGALS3 (rs11125)*	4	<0.0001	Allergic reaction	Spanish	Cornejo-García et al. (2016)
		5.1			Italian	
Penicillin and cephalosporin	HLA DQA1*01:05	2.93	5.4 × 10^−7^	Immediate hypersensitivity reactions	European	Nicoletti et al. (2021a)
	HLA DRB1*10:01	2.93	55.4 × 10^−7^			
	*TNFA–308AA*	NR	0.0046	IgE-mediated allergy	Italian	Guéant-Rodriguez et al. (2008)
Cephalosporins	HLA-B*55:02	1.76 (1.18–2.61)	0.005	Allergic reaction	Taiwanese	Wang et al. (2024b)
	HLA-C*01:02	1.36 (1.05–1.77)	0.018			
	HLA-DQB1*06:09	2.58 (1.62–4.12)	<0.001			
Penicillin	HLA-B*55:01	1.41 (1.33–1.49)	2.04 × 10^−31^	Allergic reaction	European	Krebs et al. (2020)
	HLA-DPB1*05:01	1.36	0.004	Hypersensitivity reactions	Taiwanese	Wang et al. (2024a)
	HLA-DQB1*05:01	1.54	0.03			
Flucloxacillin	HLA-A*01:01	1.86 (1.5–2.31)	1.8 × 10^−8^	Drug-induced liver injury	United Kingdom, Sweden, Netherlands, and Australia	Nicoletti et al. (2019)
	HLA-B*57:01	36.62 (26.14–51.29)	2.67 × 10^−97^			
	HLA-B*57:03	79.21 (3.37–116.1)	1.2 × 10^−6^			
	HLA-C*06:02	10.11 (7.88–12.97)	4.3 × 10^−74^			
	HLA-DQA1*02:01	4.02 (3.22–5.01)	4.5 × 10^−35^			
	HLA-DQB1*03:03	10.18 (7.77–13.34)	1.1 × 10^−63^			
	HLA-DRB1*07:01	4.02 (3.23–5.02)	3.8 × 10^−35^			
Cefaclor	HLA-DRB1*04:03	4.61 (1.51–14.09)	<0.002	Immediate hypersensitivity	Korean	Park et al. (2024)
	HLA-DRB1*14:54	3.86 (1.09–13.67)	<0.002			
	*LIMD1 (rs62242177* and *rs62242178)*	NR	5 × 10^−8^			
Anti-tuberculosis drugs						
Isoniazid, rifampicin, pyrazinamide, and ethambutol	*CYP2D6 (rs1135840)*	2.52 (1.43–4.44)	0.009	Hepatotoxicity and leukopenia	Chinese	Hu et al. (2018)
	CYP3A4*18 heterozygous genotype	3.24 (1.06–9.86)	0.034	Hepatotoxicity	Taiwanese	Lee et al. (2024)
	CYP2E1 C1/C1 + NAT2 slow acetylators (NAT2*5B/7B, *6A/6A, *6A/19, *6A/7B, *6J/7B, *7A/7B, and *7B/7B)	5.33 (1.80–15.80)	0.003	Hepatotoxicity	Chinese	An et al. (2012)
	*GSTM1 null*	2.14 (1.1–4.1)	0.02	Anti-tuberculosis drug-induced hepatotoxicity	Western Indian	Gupta et al. (2013)
	*GSTM1 and T1 null*	7.18 (1.7–32.6)	0.007			
	*GSTT1 null*	2.03 (0.9–4.4)	0.08			
	*GSTM1 null*	NR	0.007	Intensity of the anti-tuberculosis drug-induced liver injury	Brazilian	Monteiro et al. (2012)
	*GSTM1 (rs412543)*	4.44 (1.53–12.89)	0.01	Treatment-related adverse events including hepatotoxicity	Brazilian	Amorim et al. (2023)
	HLA-DQB1*05/*05	5.284 (1.134–24.615)	0.034	Liver injury	Chinese	Chen et al. (2015)
	*IL6 (rs1800796G)*	2.48 (1.40–4.40)	0.002	Hepatotoxicity	Chinese	Li et al. (2018)
	NAT2*6A	4.75 (1.8–12.55)	0.00077	Liver injury	Indonesian	Yuliwulandari et al. (2016)
	NAT2*5B, NAT2*5C, NAT2*6A, NAT2*7A, and NAT2*7B	3.45 (1.79–6.67)	1.7 × 10^−4^			
	NAT2*6A/7B	9.57 (2.72–33.62)	<0.001	Hepatotoxicity	Chinese	An et al. (2012)
	NAT2*6A/6A	5.24 (1.41–19.46)	0.013			
	*NAT2 slow acetylator*	3.64 (2.21–6.00)	0.0000002	Anti-tuberculosis drug-induced liver injury	Indonesian	Yuliwulandari et al. (2019)
	*NAT2 ultra-slow acetylator*	3.37 (2.00–5.68)	0.0000043			
	Slow acetylators (NAT2 *5/*5, *5/*6, *5/*7, *6/*6, *6/*7, *6/*14, and *7/*7)	NR	0.03	Hepatotoxicity	European, African, Latin, Asian, and Indian	Schiuma et al. (2025)
	Slow NAT2 acetylators (patients lacking NAT2*4)	8.80 (4.01–19.31)	1.53*10^−8^	Liver injury	Thai	Wattanapokayakit et al. (2016)
	Slow acetylators [rs1801280 (NAT2*5), rs1799930 (NAT2*6), rs1799931 (NAT2*7), and rs1801279 (NAT2*14)]	2.32 (0.79–6.77)		Treatment-related adverse events including hepatotoxicity	Brazilian	Amorim et al. (2023)
	Slow acetylators (NAT2 *5/*5, *5/*6, *5/*7, *6/*6, *6/*7, and *7/*7)	3.56 (1.256–10.119)		Liver injury	Mongolian	Zhang et al. (2020)
	*NR1I2 (rs7643645)*	1.64 (1.03–2.62)	0.04	Treatment failure/recurrent	Brazilian	Amorim et al. (2023)
	*rs1495741*	6.01 (3.42–10.57)	6.86E-11	Anti-tuberculosis drug-induced liver injury	Thai	Suvichapanich et al. (2019)
	*NUDT15 (rs116855232)*	4.97 (2.06–11.97)	0.003	Hepatotoxicity and leukopenia	Chinese	Hu et al. (2018)
	*PXR 63396TT*	4.575 (1.388–15.083)	0.007	Higher risk of death	Ugandan	Calcagno et al. (2019)
	*PXR 63396TT*	2.944 (1.164–7.443)	0.018	Worsening peripheral neuropathy		
	*SLCO1B1 (rs11045819)*	2.89 (1.26–6.62)	0.01	Treatment-related hepatic adverse effects	Brazilian	Amorim et al. (2023)
	*TNF-a-308G/A*	1.94 (1.04–3.63)	0.034	Anti-tuberculosis drug-induced hepatitis	Korean	Kim et al. (2012)
Isoniazid	*ASTN2 (rs117491755)*	4.37 (2.25–16.29)	1.0 × 10^−4^	Liver injury	European and Indian	Nicoletti et al. (2021b)
	CYP2E1 *1A/*1A	0.4 (1.1–12)	0.02	Hepatitis	Caucasians, Hispanic, African, South Americans, Asians, and Middle Eastern	Vuilleumier et al. (2006)
	DraI C/D (CYP2E1) and slow acetylator of NAT2 (NAT2 *5/*5, *5/*6, *5/*7, *6/*6, *6/*7, and *7/*7)	8.41 (1.54–45.76)	0.01	Hepatotoxicity	Tunisian	Ben Fredj et al. (2017)
	HLA-B*52:01	2.67 (1.63–4.37)	9.4 × 10^−5^	Liver injury	European and Indian	Nicoletti et al. (2021b)
	NAT2*5	0.69 (0.57–0.83)	0.01			
	Ultra- slow (NAT2*6/*6, *6/*7, and *7/*7)	1.89 (0.84–4.22)	0.004			
	*NAT2 (rs1041983)*	13.86 (4.3044.70)	4.754 × 10^−4^	Liver injury	Singaporean	Chan et al. (2017)
	*NAT2(rs1495741)*	0.10 (0.03–0.33)	0.004			
	*NAT2 slow acetylator*	9.98 (3.32–33.80)	8.36 × 10^−5^			
	*Rapid acetylators (NAT2∗4*, *∗12A*, *and ∗13A)*	1.26 (0.67–2.37)	0.47	Fatal treatment outcome incidence	Thai	Kasamatsu et al. (2025)
	*rs1041983 (282c > T) (NAT2)*	NR	0.002	Liver injury	Indian	Thomas et al. (2025)
	*rs1799931 (857G > A) (NAT2)*	NR	0.009			
Levofloxacin, bedaquiline, ethionamide, cycloserine, delamanid, pyrazinamide, meropenem, linezolid, and moxifloxacin	CYP2E1 C1/C1 + NAT2 slow acetylators (NAT2*5B/7B, *6A/6A, *6A/19, *6A/7B, *6J/7B, *7A/7B, and *7B/7B)	5.33 (1.80–15.80)	0.003	Central nervous system toxicity	Nigerian	Badamasi et al. (2024)
Rifampin	SLCO1B1*15	2.04 (1.05–3.96)	0.03	Liver injury	Chinese	Li et al. (2012)
Aminoglycosides						
Gentamicin	*MT-RNR1 m.1555A>G*	1.26 (1.07–1.49)	0.0058	Ototoxicity	NR	Göpel et al. (2014)
	*NOS3 (p Glu298Asp)*	NR	<0.03	Vestibular dysfunction	White	Roth et al. (2008)
Anticancer antibiotics						
Doxorubicin	*ABCC1 (rs2889517* and *rs2074087)*	0.54 (0.34–0.84)	0.006	Gastrointestinal toxicity	European American, African American, Asian, and others	Yao et al. (2014)
	*ALDH1A1 (rs3764435 and rs168351)*	1.44 (1.16–1.78)	0.0008	Hematological toxicity		
	*SLC22A16 T > C (rs714368)*	0.31 (0.12–0.75)	0.01	Neutropenia	Egyptian	Ebaid et al. (2024)
	*SLC22A16 T > C (rs714368)*	0.18 (0.07–0.5)	0.001	Leukopenia		
	*TACR1 1323C > T: TT*	2.556 (1.206–5.415)	0.0143	Nausea and vomiting	Japanese	Tsuji et al. (2021)
Doxorubicin, daunomycin, epirubicin, and idarubicin	*CBR3:GG* (with low dose, 1–250 mg/m^2^)	5.48 (1.81–16.63)	0.003	Cardiomyopathy	Hispanic, Non-Hispanic, Black, and others	Blanco et al. (2012)
	*CBR3:GG* (with low to moderate dose, 1–250; 250 mg/m^2^)	3.30 (1.41–7.73)	0.006			
Epirubicin	*GSTP1A>G*	6.4 (1.05–39.0)	0.044	Hematological toxicity	Spanish	Zárate et al. (2007)
	*GSTP1A>G*	6.5 (1.4–31)	0.018	Overall toxicities		
	*MTHFR 1298A>C*	24 (2.3–254)	0.008	Non-hematological toxicities		
	*MTHFR 1298A>C*	5.7 (1.8–17.6)	0.003	Overall toxicities		
	*MTHFR + NQO1* (Either variant)	0.36 (0.14–0.94)	0.038	Anemia	Indian	Chaturvedi et al. (2015)
	*NQO1609TT*	0.34 (0.12–0.95)	0.041			
	*NQO1609TT*	0.33 (0.12–0.88)	0.027	Grade 2–4 anemia, leukopenia, or thrombocytopenia		
Doxorubicin, daunorubicin, epirubicin, and other	*SLC28A3 (rs7853758)*	0.46 (0.20–1.08)	1.6 × 10^−5^	Cardiotoxicity	NR	Visscher et al. (2013)
	*SLC28A3 (rs885004)*	0.42 (0.16–1.10)	3.0 × 10^−5^			
	*UGT1A6 (rs17863783)*	7.98 (1.85–34.4)	2.4 × 10^−4^			
Doxorubicin and daunorubicin	*ABCA1 (rs3887137)*	2.33 (1.31–4.15)	0.0041	Cardiotoxicity	Canadian	Visscher et al. (2015)
	*ABCB4 (rs1149222)*	1.87 (1.20–2.92)	0.0054			Visscher et al. (2012)
	*ABCB11 (rs10497346)*	2.29 (1.16–4.54)	0.018			Visscher et al. (2015)
	*ABCC1 (rs4148350)*	3.44 (1.65–7.15)	0.0012			Visscher et al. (2012)
	*ABCC9 (rs11046217)*	4.48 (2.10–9.57)	7.1 × 10^−5^			Visscher et al. (2015)
	*ABCC10 (rs1214763)*	0.34 (0.15–0.75)	0.0031			
	*COL1A2 (rs42524)*	1.78 (1.11–2.88)	0.018			Visscher et al. (2015)
	*CYP2J2 (rs2294950)*	0.41 (0.19–0.90)	0.015			Visscher et al. (2015)
	*FMO2 (rs2020870)*	0.14 (0.03–0.59)	4.2 × 10^−4^			Visscher et al. (2012)
	*GPX3 (rs2233302)*	0.27 (0.11–0.65)	7.4 × 10^−4^			Visscher et al. (2015)
	*GSTM3 (rs12059276)*	0.37 (0.14–0.96)	0.027			Visscher et al. (2015)
	*HNMT (rs17583889)*	1.91 (1.21–3.02)	0.0057			Visscher et al. (2012)
	*SERPINA6 (rs10144771)*	2.23 (1.39–3.58)	9.0 × 10^−4^			Visscher et al. (2015)
	*SLC28A3 (rs7853758)*	0.31 (0.16–0.60)	1.0 × 10^−4^			Visscher et al. (2012)
	*SLC10A2 (rs9514091)*	0.43 (0.23–0.78)	0.0033			Visscher et al. (2012)
	*SLC28A3 (rs4877847)*	0.60 (0.41–0.89)	0.0092			Visscher et al. (2012)
	*SLC22A17 (rs4982753)*	0.52 (0.31–0.85)	0.0078			Visscher et al. (2015)
	*SLC22A7 (rs4149178)*	0.41 (0.21–0.77)	0.0034			
	*SLCO4C1 (rs2600834)*	2.01 (1.28–3.16)	0.0022			
	*SLCO6A1 (rs12658397)*	1.83 (1.20–2.80)	0.0048			
	*SOD2 (rs7754103)*	0.30 (0.10–0.94)	0.02			
	*SPG7 (rs2019604)*	0.39 (0.20–0.76)	0.0021			Visscher et al. (2012)
	*SULT2B1 (rs10426628)*	1.60 (1.03–2.48)	0.037			Visscher et al. (2015)
	*UGT1A6 (rs6759892)*	1.77 (1.20–2.61)	0.0038			Visscher et al. (2012)
	*XDH (rs4407290)*	0.26 (0.06–1.16)	0.035			Visscher et al. (2015)
Bleomycin	*BLMH (rs1050565GG)*	16.73 (1.78–157.15)	0.014	Pain	Chilean	Lavanderos et al. (2019)
	*CYP3A41B (rs2740574AG)*	6.87 (1.02–46.06)	0.047	Alopecia		
	*ERCC2 (rs1799793AA)*	27.00 (1.68–434.44)	0.02	Anemia		
	*ERCC2 (rs238406AA)*	5.50 (1.26–24.10)	0.024	Leukopenia		
	*ERCC2 (rs238406CA + AA)*	4.58 (1.20–17.45)	0.026			
	*ERCC2 (rs13181TG)*	10.86 (1.16–101.35)	0.036	Alopecia		
	*GSTP1(rs1695GG)*	12.25 (1.05–143.09)	0.046	Infections		
	*GSTT1 null*	17.67 (1.23–252.73)	0.034	Lymphocytopenia		
	*GSTM1* poor/intermediate genotype	NR	0.05	Anemia, neutropenia, hemorrhagic cystitis, infections, mucositis, nausea and vomiting, and cardiac, renal, or respiratory toxicities	Spanish	Altés et al. (2013)
Sulfonamides						
Co-trimoxazole	*GCLC (rs761142 TG)*	2.2 (1.4–3.7)	0.0014	Hypersensitivity	USA	Wang et al. (2012)
	*GCLC (rs761142 GG)*	3.3 (1.6–6.8)	0.001			
	HLA-A*11:01	6.97 (1.45–33.67)	0.0067	DRESS	Thai	Sukasem et al. (2020)
	HLA-B*13:01	15.20 (3.68–62.83)	7.2 × 10^−5^			
	HLA-B*15:02	5.16 (1.63–16.33)	0.0075	SJS/TEN		
	HLA-B*38:02	4.05 (1.25–13.18)	0.0249			
	HLA-B*07:02	NR	0.000001	Respiratory failure	White, Asian, and mixed	Goldman et al. (2022)
	HLA-B*13:01	8.44 (2.66–26.77)	2.94 × 10^−4^	SCARs (specifically DRESS)	Thai	Nakkam et al. (2022)
	HLA-C*03:04	4.67 (1.34–16.24)	0.0162	DRESS	Thai	Sukasem et al. (2020)
	HLA-C*07:27	43.57 (1.96–969.96)	0.0126	DRESS	Thai	Sukasem et al. (2020)
	HLA-C*07:27	27.73 (1.27–604.11)	0.0259	SJS/TEN	Thai	Sukasem et al. (2020)
	HLA-C*08:01	5.79 (1.79–18.70)	0.0049			
	HLA-C*07:02	NR	0.000018	Respiratory failure	White, Asian, and mixed	Goldman et al. (2022)
	HLA-C*08:01	8.51 (2.18–33.14)	8.60 × 10^−4^	SJS/TEN in AIDS patients	Thai	Nakkam et al. (2022)
Sulfasalazine	HLA- B*13:01	11.16 (1.98–62.85)	0.007	DRESS	Chinese	Yang et al. (2014)
	HLA- B*15:05	56.40 (3.07–1034.74)	0.041			
	HLA- B*39:01	20.14 (1.77–229.18)	0.025			
Other antibiotics						
Levofloxacin	HLA-B*13:01	4.5 (1.15–17.65)	0.043	SCARs	Chinese	Jiang et al. (2023)
	HLA-B*13:02	6.14 (1.73–21.76)	7.21 × 10^−3^			
	*HLA-Serotype B13*	17.73 (3.61–86.95)	4.85 × 10^−5^			
	HLA-DQA1*03:01	3.0 (1.5–6.1)	0.005	Liver injury	White, Black, Asian, and other	Ahmad et al. (2025)
	HLA-DQA1*03:01 or HLA-B*57:01	3.2 (1.16–8.85)	0.01			
Ciprofloxacin	HLA-B*57:01	3.1 (1.1–6.9)	0.03			
Moxifloxacin	HLA-DQA1*03:01	4.2 (1.3–13.4)	0.03			
	HLA-B*57:01	6.3 (1.4–28.2)	0.05			
	HLA-DQA1*03:01 or HLA-B*57:01	9.3 (1.5–97.4)	0.006			
Vancomycin	HLA-A*32:01	NR	<0.001	DRESS and liver injury	NR	Asif et al. (2024)
	HLA-A*32:01	NR	1 × 10^−8^	DRESS	Caucasian, Hispanic, and African American	Konvinse et al. (2019)
Clindamycin	HLA-B*15:27	55.600 (4.647–665.240)	0.0138	cADRs	Chinese	Yang et al. (2017)
	HLA-B*51:01	9.731 (2.927–32.353)	0.0018			
	HLA-B*51:01	24.000 (3.247–177.405)	0.0024	cADRs (with IV drip)		
Dapsone	HLA-B*13:01	54.00, 95% CI: 7.96–366.16	0.0001	SCARS	Thai	Tempark et al. (2017)
	HLA-B*15:02	14.00 (1.45–134.87)	0.013			
	HLA-B*13:01	60.75 (7.44–496.18)	0.0001	DRESS		
	HLA-B*13:01	40.50 (2.78–591.01)	0.007	SJS/TEN		
	HLA-B*15:02	28.00 (1.71–458.84)	0.0326			
	HLA-B*13:01	39.00 (7.67–198.21)	5.344 × 10^−7^	SCARs	Thai and Taiwanese	Satapornpong et al. (2021)
	HLA-B*13:01	36.00 (3.19–405.89)	2.165 × 10^−3^	SJS/TEN		
	HLA-B*13:01	40.50 (6.38–257.03)	1.078 × 10^−5^	DRESS		
	HLA-C*03:04	9.00 (2.17–37.38)	0.0023	SCARs		
	HLA-C*03:04	13.50 (1.71–106.56)	0.0212	SJS/TEN		
	HLA-C*03:04	7.50 (1.56–36.17)	0.0155	DRESS		
	HLA-DQB1*06:01	5.44 (1.39–21.24)	0.0258	SCARs		
	HLA-DQB1*06:01	5.83 (1.29–26.46)	0.0274	DRESS		
	HLA-DRB1*15:01	5.44 (1.39–21.24)	0.0258	SCARs		
	HLA-DRB1*15:01	10.50 (1.39–79.13)	0.0327	SJS/TEN		
Azithromycin	HLA-DQA1*03:01	3.44 (1.73, 6.47)	0.001	Liver injury	Non-Hispanic white	Conlon et al. (2024)
Minocycline	HLA-B*35:02	29.6 (7.8–89.8)	2.5 × 10^−8^	Hepatotoxicity	Caucasian	Urban et al. (2017)

Here, DRESS, drug reaction with eosinophilia and systemic symptoms; SJS, Stevens-Johnson syndrome; TEN, toxic epidermal necrolysis; SCAR, severe cutaneous adverse reactions; cADR, cutaneous adverse drug reaction; Ig, immunoglobulin; PGx, pharmacogenomics; NR, not reported; OR, odds ratio; CI, confidence interval.

### 3.2 Current evidence of PGx for antibiotic-induced hypersensitivity and adverse drug reactions

#### 3.2.1 Beta-lactam antibiotics

We identified eight studies assessing the PGx associations of genes with beta-lactam antibiotics for DIHRs and other adverse effects. These studies primarily investigated the genetic associations with the DIHRs, with only one study examining the genetic link to flucloxacillin-induced liver injury (Wang et al., 2024b; Wang et al., 2024a; Park et al., 2024; Nicoletti et al., 2021a; Nicoletti et al., 2019; Krebs et al., 2020; Guéant-Rodriguez et al., 2008; Cornejo-García et al., 2016). Cornejo-García et al. (2016) proposed that *LGALS3* could be a potential genetic predictor of immediate drug reactions and reported that *rs11125* of *LGALS3* (odds ratio, OR = 5.1 in the Italian population (*p* < 0.0001)) was strongly associated with beta-lactam (BL)-induced allergy. Mast cells release tumor necrosis factor-α (*TNF-α*) via an immunoglobulin E (IgE)-dependent mechanism. *TNFA–308G>A* is part of the extended haplotype *HLA-A1-B8-DR3-DQ2* and influences the expression of the gene. Guéant-Rodriguez et al. (2008) evaluated this variant in relation to IgE-mediated reactions to BLs and reported its association with the BL-induced immediate allergic reactions. They observed that individuals carrying the *–308AA* genotype exhibited significantly higher specific IgE serum levels compared to those with the *–308GA/GG* genotype (*p* = 0.0046) (Guéant-Rodriguez et al., 2008).

Other studies aimed to evaluate the association between different *HLA* genes and DIHRs. Nicoletti et al. (2021a) identified HLA-DRB1*10:01 (OR = 2.93; *p* = 5.4 × 10^−7^) as a risk factor for immediate reaction with BLs even without the HLA-DQA1*01:05 allele (OR = 2.93, *p* = 5.4 × 10^−7^). Park et al. (2024) identified *LIMD1 (rs62242177* and *rs62242178)* (significance level 5 × 10^−8^), HLA-DRB1*04:03 (OR = 4.61, 95% confidence interval (CI): 1.51–14.09, *p* < 0.002), and HLA-DRB1*14:54 (OR = 3.86, 95% CI: 1.09–13.67, *p* < 0.002) as potential factors influencing susceptibility to cefaclor-induced type I hypersensitivity. Krebs et al. (2020) provided robust evidence of HLA-B *55:01 (OR = 1.41; 95% CI: 1.33–1.49, *p* = 2.04 × 10^−31^) being associated with the occurrence of penicillin allergy through a genome-wide study. Wang et al. (2024a) reported HLA-DPB1*05:01 (OR = 1.36, *p* = 0.004) and HLA-DQB1*05:01 (OR = 1.54, *p* = 0.03) to be significantly linked with penicillin allergy among Taiwanese. For cephalosporin, on the other hand, Wang et al. (2024b) identified HLA-DQB1*06:09 (OR = 2.58, 95% CI: 1.62–4.12, *p* < 0.001), HLA-C*01:02 (OR = 1.36, 95% CI: 1.05–1.77, *p* = 0.018), and HLA-B*55:02 (OR = 1.76, 95% CI: 1.18–2.61, *p* = 0.005) alleles to be linked with cephalosporin-induced allergy. Nicoletti et al. (2019) performed a genome-wide association study and reported the following associations with flucloxacillin-induced liver injury: HLA-B *57:01 (allelic OR = 36.62, 95% CI: 26.14–51.29, *p* = 2.67 × 10^−97^), HLA-A *01:01(OR = 1.86, 95% CI: 1.5–2.31, *p* = 1.8 × 10^−8^), HLA-C*06:02 (OR = 10.11, 95% CI: 7.88–12.97, *p* = 4.3 × 10^−74^), HLA-B *57:03 (OR = 79.21, 95% CI: 3.37–116.1, *p* = 1.2 × 10^−6^), HLA-DQB1*03:03 (OR = 10.18, 95% CI: 7.77–13.34, *p* = 1.1 × 10^−63^), HLA-DRB1*07:01 (OR = 4.02, 95% CI: 3.23–5.02, *p* = 3.8 × 10^−35^), HLA-DQA1*02:01 (OR = 4.02, 95% CI: 3.22–5.01, *p* = 4.5 × 10^−35^). They also stated no association of HLA-B*57 with drug-induced liver injury (DILI) for other isoxazolyl penicillin or amoxicillin (Nicoletti et al., 2019).

These studies are population-based and involve varying sample sizes. Consequently, studies with smaller case numbers may either underestimate or overestimate the findings. Therefore, further evaluation with a larger sample size was encouraged for better understanding, rationalization, and integration of that information in clinical practice.

#### 3.2.2 Aminoglycosides

We identified at least four studies that associated aminoglycoside-induced ototoxicity with *MT-RNR1* mutations (Roth et al., 2008; Fischel-Ghodsian et al., 1997; Lu et al., 2010; Göpel et al., 2014). Göpel et al. (2014), using a multivariable logistic regression, demonstrated treatment with aminoglycosides in *m.1555A>G*-carriers was associated with the failed hearing screening (OR = 1.26; 95% CI: 1.07–1.49; *p* = 0.0058). They also observed the *m.1555A>G* mutation in all the mothers of the children carrying the *m.1555A>G* mutation, which was absent in the mothers of the non-carrier children of the *m.1555A>G* mutation. They suggested antenatal screening of the *m.1555A>G* mutation through maternal genotyping of pregnant women with preterm labor may potentially be a rational approach to identifying infants with an increased risk of permanent hearing loss (Göpel et al., 2014). Lu et al. (2010) observed *745A>G*, *792C>T*, *801A>G*, *839A>G*, *856A>G*, *1027A>G*, *1192C>T*, *1192C>A*, *1310C>T*, *1331A>G*, *1374A>G*, *and 1452T>C* variants to confer increased sensitivity to nonsyndromic deafness or ototoxic drugs. Bilateral and sensorineural hearing loss was exhibited in 65 Chinese individuals who carried the *1555A>G* mutation (Lu et al., 2010). Fischel-Ghodsian et al. (1997) explored the irreversible sensorineural hearing loss (SNHL) with the use of aminoglycosides (streptomycin, gentamicin, kanamycin, amikacin, and neomycin) due to *m.1555A > G* variants in mitochondrial 12S RNA and observed the presence of polymorphism in 17% of the total population having SNHL after aminoglycoside exposure, and among them, more than half had a family history of SNHL with aminoglycosides. Therefore, they recommended clinical screening and appropriate familial evaluation to avoid associated ototoxicity (Fischel-Ghodsian et al., 1997). Roth et al. (2008) stated that carriers of risk alleles of *NOS3 (p.Glu298Asp)*, *GSTZ1 (p.Lys32Glu)*, and *GSTP1 (p.Ile105Val)* are relevant for the elevated risk of vestibular dysfunction with gentamicin (*p* < 0.03).

#### 3.2.3 Sulfonamides

We identified at least five studies that correlated co-trimoxazole/sulfamethoxazole/trimethoprim with genetic association (Nakkam et al., 2022; Goldman et al., 2022; Alfirevic et al., 2009; Wang et al., 2012; Sukasem et al., 2020). Similarly, one such study explored the genetic association with sulfasalazine-induced ADRs (Yang et al., 2014). Nakkam et al. (2022) reported that the HLA-B*13:01 allele was significantly associated with co-trimoxazole-induced SCARs, particularly DRESS (OR = 8.44, 95% CI: 2.66–26.77, *p* = 2.94 × 10^−4^). Additionally, the HLA-C*08:01 allele was observed to have a significant association with SJS/TEN induced by co-trimoxazole in HIV/AIDS patients [OR of 8.51, 95% CI: 2.18–33.14, *p* = 8.60 × 10^−4^] (Nakkam et al., 2022). Goldman et al. (2022) evaluated respiratory failure with trimethoprim/sulfamethoxazole and *HLA* and identified HLA-B *07:02 (*p* = 0.000001) and HLA-C *07:02 (*p* = 0.000018) to be significantly associated with the increased risk of respiratory failure. However, Alfirevic et al. (2009) stated that *MHC* polymorphisms were not a major predisposing factor for co-trimoxazole hypersensitivity, although a minor contribution cannot be ruled out. For sulfamethoxazole (SMX)-induced hypersensitivity in HIV/AIDS patients, Wang et al. (2012) reported that *GCLC (rs761142 T>G)* was significantly associated with hypersensitivity induced by SMX (adjusted *p*-value = 0.045). In a replicated cohort with 249 patients, the result was replicated (*p* = 0.025). For the combined cohort, homozygous and heterozygous carriers of the minor *G* allele were recorded for an increased risk of hypersensitivity (*GT* vs *TT*, OR = 2.2, 95% CI: 1.4–3.7, *p* = 0.0014; *GG* vs. *TT*, OR = 3.3, 95% CI: 1.6–6.8, *p* = 0.0010). Each minor allele copy increased the risk of developing hypersensitivity 1.9-fold (95% CI: 1.4–2.6, *p* = 0.00012) (Wang et al., 2012). Sukasem et al. (2020) identified HLA-C*08:01 (OR = 5.79, 95% CI: 1.79–18.70, *p* = 0.0049) and HLA-B*15:02 (OR = 5.16, 95% CI: 1.63–16.33, *p* = 0.0075) alleles as significantly associated with SJS/TEN induced by co-trimoxazole, and the HLA-B*13:01 allele was significantly linked to co-trimoxazole-induced DRESS (OR = 15.20, 95% CI: 3.68–62.83, *p* = 7.2 × 10^−5^). Additionally, significantly high frequency of HLA-B*13:01-C*03:04 (OR = 14.53, 95% CI: 3.74–56.47, *p* = 1.8 × 10^−4^) and HLA-A*11:01-B*15:02 (OR = 6.00, 95% CI: 1.72–20.88, *p* = 0.0074) haplotypes were observed in the group of co-trimoxazole-induced DRESS and SJS/TEN, respectively (Sukasem et al., 2020).

In the Chinese Han population, Yang et al. (2014) explored sulfasalazine-induced DRESS and identified HLA-B*13:01 as a potential biomarker for increasing the risk of DRESS since the distribution of the HLA-B*13:01 allele was significantly higher in sulfasalazine-induced DRESS patients than in sulfasalazine-tolerant patients (OR = 13.00, 95% CI: 1.76–95.80, *p* = 0.004) (Yang et al., 2014).

#### 3.2.4 Anti-tuberculous drugs

We identified at least 25 studies evaluating the PGx associations of different genes with anti-tuberculous drug (ATD)-induced adverse effects (Amorim et al., 2023; An et al., 2012; Badamasi et al., 2024; Ben Fredj et al., 2017; Calcagno et al., 2019; Chan et al., 2017; Chen et al., 2015; Gupta et al., 2013; Hu et al., 2018; Kasamatsu et al., 2025; Kim et al., 2012; Lee et al., 2024; Li et al., 2012; Li et al., 2018; Monteiro et al., 2012; Nicoletti et al., 2021b; Schiuma et al., 2025; Suvichapanich et al., 2019; Thomas et al., 2025; Vuilleumier et al., 2006; Wattanapokayakit et al., 2016; Yamada et al., 2010; Yuliwulandari et al., 2019; Yuliwulandari et al., 2016; Zhang et al., 2020). Of these, the study by Li et al. evaluated the association of ATDs in pediatric patients and reported a striking difference in the allele distribution of *rs1800796* in the *IL6* gene between the control and case groups, and the *G* allele of *rs1800796* was linked with an elevated risk for anti-tuberculosis drug-induced hepatotoxicity (OR = 2.48, 95% CI: 1.40–4.40, *p* = 0.002). After Bonferroni correction, no significant difference was observed in the allele and genotype distributions of the other SNPs in the *IL6*, *XO*, and *NOS2* genes between the control and case groups (Li et al., 2018). Three studies evaluated the association of *GSTM1* and *GSTT1* with ATDs. They reported that the homozygous null mutation of the *GSTM1* gene, either alone or in combination with *T1*, was significantly associated with anti-tuberculosis drug-induced hepatotoxicity (*p* < 0.02 and *p* < 0.007, respectively); one study further reported that the *GSTM1* polymorphism *(rs412543)* (p = 0.01) was linked to an elevated risk of treatment-related adverse events, including hepatotoxicity. Conversely, another study found no significant role of the *GSTM1* and *GSTT1* null genotypes in anti-tuberculosis drug-induced liver injury, although there was evidence that *GSTM1* polymorphisms may be related to the intensity of toxicity (*p* = 0.007) (Amorim et al., 2023; Gupta et al., 2013; Monteiro et al., 2012).

Yuliwulandari et al. (2019) found that the *NAT2* slow-acetylator phenotype was significantly associated with the risk of AT-DILI (*p* = 2.7 × 10^−7^, OR = 3.64, 95% CI: 2.21–6.00). The *NAT2* ultra-slow acetylator showed an even stronger association with *AT-DIL*I risk in the subgroup analysis (*p* = 4.3 × 10^−6^, OR = 3.37, 95% CI: 2.00–5.68). In the Thai population, Suvichapanich et al. (2019) reported that the *A* allele of *rs1495741*, the top SNP in the intergenic region of *NAT2* and *PSD3*, was significantly associated with anti-tuberculosis drug-induced liver injury (ATDILI) (OR = 6.01, 95% CI: 3.42–10.57, *p* = 6.86E-11), identifying that *NAT2* ultra-slow acetylator as the most important risk factor for ATDILI. In the Indian population, Thomas et al. (2025) observed that allele *T (rs1041983)* (*p* = 0.002) and allele *A (rs1799931)* (*p* = 0.009) were associated with an elevated risk of drug-induced liver injury in patients receiving anti-tubercular drugs, compared to allele *C* and allele *G*, respectively. Schiuma et al. (2025) reported that NAT2*5/*5, *5/*6, *5/*7, *6/*6, *6/*7, *6/*14, and *7/*7 (grouped as the slow-acetylator phenotype) were linked to an increased likelihood of toxic liver disease during treatment with ethambutol and isoniazid/pyrazinamide/rifampin in individuals with tuberculosis (*p* = 0.03), compared to NAT2*1/*5, *1/*6, and *1/*7 (grouped as intermediate acetylator and rapid acetylator phenotypes). Three additional studies confirmed that slow *NAT2* acetylators are a risk factor for ATDILI. Specifically, NAT2*6 was associated with an increased risk (OR = 4.75, 95% CI: 1.80–12.55, *p* = 0.00077), while no significant association was observed for NAT2*5 or *7. On the contrary, NAT2*4 was associated with a decreased risk of drug-induced liver injury (*p* = 1.8 × 10^−6^, OR = 0.2, 95% CI: 0.1–0.39); compared to intermediate or rapid acetylators (NAT2*4, NAT2*12A, and NAT2*13), slow acetylators due to *NAT2* genotypes (NAT2*5B, NAT2*5C, NAT2*6A, NAT2*7A, and NAT2*7B) exhibited a higher risk of liver injury (*p* = 1.7 × 10^−4^, OR = 3.45, 95% CI: 1.79–6.67). Overall, the slow-acetylator type due to the polymorphism of *NAT2* was considered a risk factor for ATDILI (OR = 3.56, 95% CI: 1.256–10.119), and slow *NAT2* acetylators (patients lacking NAT2*4) showed a significant association with ATDILI risk (OR = 8.80; 95% CI = 4.01–19.31, *p* = 1.53 × 10^−8^) (Wattanapokayakit et al., 2016; Yuliwulandari et al., 2016; Zhang et al., 2020). In patients with tuberculosis, Kasamatsu et al. (2025) observed that rapid acetylators due to NAT2 polymorphism had a 1.26-fold higher incidence of fatal treatment outcomes (95% CI: 0.67–2.37) compared to intermediate acetylators.

Hu et al. (2018) reported an increased risk of leukopenia and hepatotoxicity associated with *CYP2D6 rs1135840* and *NUDT15 rs116855232*, with ORs of 2.52 (95% CI: 1.43–4.44, *p* = 0.009) and 4.97 (95% CI: 2.06–11.97, *p* = 0.003), respectively. For multidrug-resistant tuberculosis treatment, Badamasi et al. (2024) reported a significant association between CNS toxicity and the dominant model of inheritance for the crude model (*p* = 0.024; OR = 3.57; 95% CI: 1.18–10.76) and the adjusted model (*p* = 0.031, OR = 3.92, 95% CI: 1.13–13.58). They reported that the *AT + TT* genotype of *IL8 (rs4073*) is associated with a 3.92-fold increased risk of CNS toxicity compared to the *AA* genotype (Badamasi et al., 2024).

Apart from the *GSTM1* association as mentioned earlier, Amorim et al. (2023) also explored other genetic associations and stated that *NAT2* slow acetylator status was linked with an increased risk of treatment-related adverse events, including hepatotoxicity, compared with rapid acetylator (OR = 2.32, 95% CI: 0.79–6.77). Treatment failure or recurrence was more likely among *NAT2* rapid acetylators. Similarly, *SLCO1B1* (*p* = 0.01) was linked with an elevated risk of treatment-related adverse events, including hepatotoxicity. Polymorphisms in *NR1I2* were associated with decreased risk of adverse effects but increased risk of failure/recurrence (p = 0.04). Although in whole exome sequencing, hepatotoxicity was associated with a polymorphism in *VTI1A*, and the genes *METTL17* and *PRSS57*, but none achieved genome-wide significance (Amorim et al., 2023). Calcagno et al. (2019) reported that *NAT2 (rs1799930)*, *SLCO1B1 (rs4149032)*, and *PXR (rs2472677)* variants affected isoniazid exposure. Genotype *TT (rs2472677)* was linked with an elevated peripheral nervous system disease (*p* = 0.018) and elevated death risk (*p* = 0.007) with treatment with ethambutol, isoniazid, efavirenz, and rifampin in people with HIV and tuberculosis compared with genotypes *CC* and *CT*.

Although univariate analyses by Chen et al. (2015) and Chan et al. (2017) found no statistically significant association between ATDILI and the frequency of *HLA-DQB1* genotypes, multivariate analysis revealed that individuals carrying two DQB1*05 alleles had a higher risk of ATDILI compared to the control group (OR = 5.28 adjusted for use of liver-protective drugs and weight 10/88 VS 2/88, 95% CI: 1.134–24.615, *p* = 0.034). Regardless of the presence of pre-existing liver disease, the heterozygous CYP3A4*18 genotype was associated with anti-tuberculosis drug-induced hepatotoxicity (ATDH) in a study by Lee et al. (2024) (OR: 3.24, 95% CI: 1.06–9.86). Although among the subjects without having liver disease, CYP3A4*18 heterozygotes were observed to have a significantly higher risk of ATDH (OR: 9.10, 95% CI: 1.56–53.16), in subjects with previous liver disease, CYP3A4*18 heterozygotes had a lower risk of ATDH (OR: 0.21, 95% CI: 0.05–0.98) (Lee et al., 2024). The frequency of -*308AG/AA* carriers was found to be significantly higher in ATD-induced hepatitis patients than the ATD-tolerant patients (*p* = 0.034, OR = 1.94; 95% CI = 1.04–3.64) and the frequency of the A allele significantly differed between the two groups (*p* = 0.018, OR 1.95, 95% CI = 1.11–3.44). These results indicated that the *TNFA-308G/A* polymorphism was significantly associated with ATDH (Kim et al., 2012). An et al. (2012) deemed slow acetylators due to *NAT2* genotypes (particularly, NAT2*6A/7B and NAT2*6A/6A) risk factors for drug-induced hepatotoxicity (DIH) (OR = 9.57; *p* < 0.001) for NAT2*6A/7B; OR 5.24 (*p* = 0.02) for NAT2*6A/6A). Although the *CYP2E1* genotype was not significantly linked with the development of anti-tuberculosis DIH, the combination of the *CYP2E1 C1/C1* genotype and the *NAT2* genotype of slow acetylator was observed to increase the risk of anti-tuberculosis (OR = 5.33; *p* = 0.003) compared to the combination of the *NAT2* rapid acetylator genotype paired with either a *C1/C2* or *C2/C2* genotype (An et al., 2012).

Six of the studies evaluated PGx’s association with the adverse effects of isoniazid alone. Chan et al. (2017), on the Singaporean population, performed a study and identified a significant association of two SNPs of *NAT2* (rs1041983 and rs1495741) and *NAT2* slow acetylators with isoniazid-induced liver injury (OR = 13.86, 95% CI: 4.30–44.70; OR = 0.10, 95% CI = 0.03–0.33 and OR = 9.98, 95% CI = 3.32–33.80, respectively). They also stated a model based on clinical and NAT2 acetylator status resulted in much better prediction for isoniazid-induced liver injury compared to a clinical model alone (area under the receiver operating characteristic curve = 0.863 vs. 0.766, respectively, *p* = 0.027) (Chan et al., 2017). A genome-wide association study by Nicoletti et al. identified *rs117491755* in *ASTN2* as being significantly associated with DILI in European patients only. HLA-B*52:01 was also found to be significant (OR = 2.67, 95% CI = 1.63–4.37, *p* = 9.4 × 10^−5^). The frequency of NAT2*5 was lower for cases (OR = 0.69, 95% CI = 0.57–0.83, *p* = 0.01). NAT2*6 and NAT2*7 were relatively common, homozygotes for NAT2*6 and/or NAT2*7 being enriched in cases (OR = 1.89, 95% CI = 0.84–4.22, *p* = 0.004). They reported that *HLA* genotypes made a minimal contribution to ATDILI and that the contribution of *NAT2* was complex. However, their findings were consistent with previous studies when considering differences in metabolic effects between NAT2*5, NAT2*6, and NAT2*7 alleles (Nicoletti et al., 2021b). Two separate studies reported that *NAT2* and C*YP2E1* variants were not associated an increased risk of isoniazid-induced hepatotoxicity when analyzed independently; however, Vuilleumier et al. found that compared with other *CYP2E1* genotypes, a significant association between the CYP2E1 *1A/*1A genotype and isoniazid-induced elevated liver enzymes, including hepatitis (OR: 3.4; 95% CI:1.1–12; *p* = 0.02), and a non-significant trend for isoniazid induced hepatotoxicity was also recorded (OR: 5.9; 95% CI: 0.69–270; *p* = 0.13). Similarly, Ben Fredj et al. stated that a combined analysis of the polymorphism in the *NAT2/CYP2E1* gene revealed that individuals with both DraI *C/D (CYP2E1)* and slow acetylator *(NAT2)* genotypes have an elevated risk of isoniazid-induced hepatotoxicity as compared to other combined *NAT2/CYP2E1* genotype profiles (OR: 8.41, *p* = 0.01, 95% CI: 1.54–45.76) (Ben Fredj et al., 2017; Vuilleumier et al., 2006). Yamada et al. (2010) found no association between isoniazid-induced hepatotoxicity SNPs and haplotypes at *CES2* and *CES1/CES4*.

Li et al. (2012) evaluated the PGx association of rifampin and identified an association between SLCO1B1*15 and the increased risk of drug-induced liver injury (*p* = 0.03, OR = 2.04, 95% CI: 1.05–3.96). No such association was found for SLCO1B1*5 and *1.

#### 3.2.5 Anticancer antibiotics

We identified at least 11 studies assessing the association of genes with the adverse effects of anthracyclines (Chaturvedi et al., 2015; Yao et al., 2014; Visscher et al., 2013; Visscher et al., 2015; Nyangwara et al., 2024; Ebaid et al., 2024; Visscher et al., 2012; Robinson et al., 2019; Zárate et al., 2007; Blanco et al., 2012; Tsuji et al., 2021). Five of them were on pediatric patients. Among those, Robinson et al. (2019) reported that *G6PD* deficiency did not have any effect on the hemolytic toxicities with daunorubicin during the induction treatment for acute lymphoblastic leukemia (*p* = 0.73). Blanco et al. (2012) observed the exposure of low-to-moderate doses of anthracyclines in individuals carrying the variant *A* allele *(CBR1:GA/AA* and/or *CBR3:GA/AA)* did not raise the risk of cardiomyopathy, but with similar doses, an increased risk of cardiomyopathy was observed in individuals with the *CBR3 V244M* homozygous *G* genotypes *(CBR3:GG)* compared to the individuals with the *CBR3:GA/AA* genotypes unexposed to anthracyclines (OR = 5.48; *p* = 0.003) and exposed to low-to-moderate doses of anthracyclines (OR = 3.30; *p* = 0.006). High doses of anthracyclines, irrespective of *CBR* genotype status, were associated with increased cardiomyopathy risk (Blanco et al., 2012). Visscher et al. identified a highly significant association with a synonymous coding variant, *rs7853758 (L461L)*, in the *SLC28A3* gene with anthracycline-induced cardiotoxicity in children (OR = 0.35; *p* = 1.8 × 10^−5^, single marker test). Additionally, other significant associations with protective and risk variants in other genes, including *SLC28A1*, *ABCB1*, *ABCB4*, and *ABCC1*, were present. For safer treatment options, combining genetic risk profiles may be considered (Visscher et al., 2012). In this replication cohort, Visscher et al. confirmed the association of *rs17863783* (*UGT1A6*) and anthracycline-induced cardiotoxicity (*p* = 0.0062, OR = 7.98). Additionally, evidence for the association of *rs885004* (p = 0.058, OR 0.42) and *rs7853758* (p = 0.058, OR 0.46) in *SLC28A3* was reported (combined *p* = 3.0 × 10^−5^ and *p* = 1.6 × 10^−5^, respectively). Unlike a previously constructed model for prediction, the improved prediction model constructed utilizing the replicated genetic variants alongside the clinical factors discriminated significantly better among cases and controls against only clinical factors, both in the original (AUC 0.77 vs. 0.68, p = 0.0031) and replication cohort (AUC 0.77 vs. 0.69, p = 0.060) (Visscher et al., 2013). In this study, Visscher et al. identified significant associations of *SLC22A7 (rs4149178*, *p* = 0.0034) and *SLC22A17 (rs4982753*, *p* = 0.0078*)* with anthracycline-induced cardiotoxicity in both discovery and replication cohort. Additionally, evidence was found for *SULT2B1* and several other genes related to oxidative stress (Visscher et al., 2015).

Yao et al. (2014) observed in breast cancer patients that *rs3764435* and *rs168351* (*ALDH1A1*) were significantly associated with hematological toxicity (OR = 1.44, 95% CI: 1.16–1.78, *p* = 0.0008), and *rs2889517* and *rs2074087* (*ABCC1*) were significantly associated with gastrointestinal toxicity (OR = 0.54, 95% CI: 0.34–0.84, *p* = 0.006). Nyangwara et al. (2024), in a study on Zimbabwean breast cancer patients, found no significant association between doxorubicin-induced cardiotoxicity and *SLC28A3 (rs7853758*, *p* = 0.408*)*, UGT1A6*4 (rs17863783, *p* = 0.354*)*, or *RARG (rs2229774*, *p* = 0.471*)*. Ebaid et al. (2024), in Egyptian breast cancer patients, reported that carriers of *CBR1 C>T (rs20572)* had significantly higher doxorubicin concentrations, but no significant association with hematological toxicity was observed. On the contrary, although no significant effect of *SLC22A16 T>C (rs714368)* on the plasma concentration was observed, it was significantly correlated with a lower risk of neutropenia (OR 0.31, 95% CI = 0.12–0.75, *p* = 0.01) and leucopenia (OR 0.18, 95% CI = 0.07–0.5, *p* = 0.001). Doxorubicin-related cardiotoxicity was associated with the cumulative doxorubicin dose (OR = 0.238, *p* = 0.017), but not with any of the two SNPs examined (Ebaid et al., 2024). Tsuji et al. (2021) reported that in breast cancer patients receiving triplet antiemetic combination regimens, *ABCB1 2677G>T/A* was not predictive of the antiemetic response. However, an association was observed between the *TACR1 1323C>T* polymorphism and complete response in the acute phase.

Among Indian breast cancer patients treated with 5-fluorouracil, epirubicin/methotrexate/adriamycin, and cyclophosphamide regimens, Chaturvedi et al. (2015) observed that grade 2–4 toxicity (anemia, leucopenia, or thrombocytopenia) was significantly associated with *NQO1609TT* (OR = 0.33, 95% CI: 0.12–0.88, *p* = 0.027). Further analysis for anemia found a significant association with *NQO1609TT* (OR = 0.34; 95% CI: 0.12–0.95; *p* = 0.041) and the combination of *MTHFR + NQO1* (either variant) (OR = 0.36; 95% CI = 0.14–0.94; *p* = 0.038) (Chaturvedi et al., 2015). For breast cancer adjuvant therapy with anthracycline (epirubicin), Zárate et al. (2007) found that hematological GIII-IV toxicity was associated with *GSTP1* polymorphism (*p* = 0.044, hazard ratio, HR = 6.4, 95% CI: 1.05–39). Evaluation of non-hematological toxicities revealed increased and significant HR for GIII-IV toxicities in the *MTHFR-1298 AC + CC* group (HR = 24, 95% CI = 2.3 to 254, *p* = 0.008). They identified *GSTP1* and *MTHFR-1298A>C* polymorphisms as independent risk factors regarding overall toxicities (Zárate et al., 2007). Two studies establishing a genetic association with bleomycin-induced ADRs were selected for the study. The first one, by Altés et al. (2013), explored the use of bleomycin in Hodgkin lymphoma and found that the carrier of *GSTM1* extensive or ultrahigh activity was linked to a decreased risk of grade III/IV toxicity development (*p* = 0.05), but with efficacy analysis, they concluded that compared to PGx determinants, clinical determinants could be more relevant for the Hodgkin lymphoma treatment. The other study explored the genetic association of toxicities with the bleomycin-containing regimen in Chilean testicular cancer patients and emphasized the need of PGx implementations for severe ADR prediction based on some robust genetic associations, including *ERCC2 (rs1799793AA)* and anemia (OR = 27.00, 95% CI = 1.68–434.44, *p* = 0.020), *ERCC2 (rs238406AA)* and leukopenia (OR = 5.50, 95% CI = 1.26–24.10, *p* = 0.024), *GSTT1 null* and lymphocytopenia (OR = 17.67, 95% CI = 1.23–252.73, *p* = 0.034), *CYP3A41B (rs2740574GG)* and alopecia (OR = 6.87, 95% CI = 1.02–46.06, *p* = 0.047), *BLMH (rs1050565)* and pain (OR = 16.73, 95% CI = 1.78–157.15, *p* = 0.014) and *GSTP1 (rs1695GG)* and infections (OR = 12.25, 95% CI = 1.05–143.09, *p* = 0.046) (Lavanderos et al., 2019).

#### 3.2.6 Other antibiotics

A study of genetic association of levofloxacin-induced SCARs in the Chinese population by Jiang et al. revealed that compared to levofloxacin-tolerant patients, significantly higher frequencies of HLA-B*13:01 (OR: 4.50, 95% CI: 1.15–17.65, *p* = 0.043), HLA-B*13:02 (OR: 6.14, 95% CI: 1.73–21.76, *p* = 0.0072), and serotype B13 (OR: 17.73, 95% CI: 3.61–86.95, *p* = 4.85 × 10^−5^) were observed in patients with levofloxacin-induced SCARs. They proposed prospective screening or alternative therapy that may benefit the patient in concern (Jiang et al., 2023). Ahmad et al. (2025) found a significant association with HLA-DQA1*03:01 and HLA -B*57:01 for DILI induced by fluoroquinolones (ciprofloxacin, levofloxacin, and moxifloxacin). Details of the specific ORs are presented in Table 1.

Of the included studies, we identified two studies that evaluated the association of *HLA* with vancomycin-induced adverse effects, such as liver injury and DRESS. Asif et al. (2024) reported that HLA-A*32:01 was associated with vancomycin-induced liver injury and DRESS (*p* < 0.001). Konvinse et al. (2019) noted that the carriage of the HLA-A*32:01 allele is significantly associated (*p* = 1 × 10^−8^) with the development of DRESS induced by vancomycin.

Yang et al. (2017) evaluated the genetic association with clindamycin-induced cADRs in the Chinese population and observed that compared to the control and clindamycin-tolerant groups, the frequency of HLA-B*51:01 was significantly higher in the case group. They identified HLA-B*51:01 as a risk allele for clindamycin-related cADRs in the Han Chinese population, particularly with clindamycin administration via an intravenous drip (OR = 24.00, 95% CI: 3.25–177.41, *p* = 0.0024). HLA-B*15:27 was also found to have a link with clindamycin-induced cADRs (OR = 55.60, 95% CI: 4.647–665.24, *p* = 0.0046, pc = 0.0184) (Yang et al., 2017).

Urban et al. (2017) explored the genetic link with minocycline hepatotoxicity and noted HLA-B*35:02 to have a significant association with the risk for minocycline-induced liver injury (OR: 29.6, 95% CI: 7.8–89.8, *p* = 2.5 × 10^−8^). Sequence-based *HLA* typing verified this association (Urban et al., 2017).

Two of the included studies explored the PGx association of dapsone-induced SCARs. Tempark et al. (2017) reported that the HLA-B*13:01 allele had a significant association with SCARs induced by dapsone compared to the dapsone-tolerant controls (OR: 54.00, 95% CI: 7.96–366.16, *p* = 0.0001) and the general population (OR: 26.11, 95% CI: 7.27–93.75, *p* = 0.0001). Additionally, HLA-B*13:01 was found to be associated with dapsone-induced DRESS (OR: 60.75, 95% CI: 7.44–496.18, *p* = 0.0001) and SJS-TEN (OR: 40.50, 95% CI: 2.78–591.01, *p* = 0.0070) in non-leprosy Thai patients (Tempark et al., 2017). Of all *HLA* alleles, Satapornpong et al. (2021) reported that only the HLA-B*13:01 allele was significantly associated with dapsone-induced SCARs (OR = 39.00, 95% CI: 7.67–198.21, *p* = 5.3447 × 10^−7^), DRESS (OR = 40.50, 95% CI: 6.38–257.03, *p* = 1.0784 × 10^−5^), and SJS-TEN (OR = 36.00, 95% CI: 3.19–405.89, *p* = 2.1657 × 10^−3^) compared with dapsone-tolerant controls. The HLA-B*13:01 allele was also strongly associated with dapsone-induced SCARs among the Taiwanese population (OR = 31.50, 95% CI: 4.80–206.56, *p* = 2.5519 × 10^−3^) and Asians (OR = 36.00, 95% CI = 8.67–149.52, *p* = 2.8068 × 10^−7^) (Satapornpong et al., 2021). Compared to the control population, Conlon et al. (2024) observed a significant association with HLA-DQA1*03:01 for azithromycin-induced liver injury (OR = 3.44, 95% CI: 1.73, 6.47, *p* = 0.001) and recommend further exploration for a comprehensive understanding of the mechanism involved and clinical role (Conlon et al., 2024).

### 3.3 Current state of PGx-based clinical annotations and drug labels for antibiotics

We used the PharmGKB clinical annotations to determine the current PGx evidence level for the variants and genes involved in the safety and effectiveness of the antibiotics. Based on variant annotations and incorporating available variant-specific prescribing guidelines and FDA-approved drug labels, these annotations provide information on the drug–variant pairs. Following a scoring system, these annotations are then assigned a level of evidence ranging from level-4 (unsupported) to level-1A (high) (PharmGKB, 2025a; Whirl-Carrillo et al., 2021). Our search across PharmGKB revealed clinical annotations for at least 36 antibiotic drugs, each with various variants of at least 85 genes. These annotations are presented in Tables 2, 3.

**TABLE 2:** Current PGx-based clinical annotations of various antibiotic–gene pairs with the PharmGKB level of evidence.

Drug	Gene	Variant	Clinical annotation	Level of evidence
Amoxicillin	*HLA-B*	HLA-B*18:01	Toxicity	3
	*HLA-DQB1*	*rs9274407*	Toxicity	3
Ceftriaxone	*ABCC2*	*rs2273697*	Metabolism/PK	3
	*ABCG2*	*rs13120400*	Metabolism/PK	3
Cefotaxime	*SLC22A8*	*rs11568482*	Metabolism/PK	3
Erythromycin	*ABCC2*	*rs717620*	other	3
	*CYP3A4*	*rs35599367*	other	3
Amikacin	*MT-RNR1*	*rs267606617*	Toxicity	1A
Neomycin	*MT-RNR1*	*rs267606617*	Toxicity	1A
Gentamicin	*MT-ND1, MT-RNR1*	*rs267606617*, *rs267606618*, *rs267606619*, and *rs28358569*	Toxicity	1A
Kanamycin	*MT-RNR1*	*rs267606617*, *rs267606618*, and *rs267606619*	Toxicity	1A
Streptomycin	*MT-RNR1*	*rs267606617*, *rs267606618*, and *rs267606619*	Toxicity	1A
	*MT-RNR1*	*rs28358569* and *rs1556422499*	Toxicity	3
	*GSTM1*	*GSTM1 non-null* and *GSTM1 null*	Toxicity	4
	*GSTT1*	*GSTT1 non-null* and *GSTT1 null*	Toxicity	4
Tobramycin	*MT-RNR1*	*rs267606617, rs267606619*	Toxicity	1A
Ciprofloxacin	*G6PD*	*G6PD B (reference)*, *G6PD Mediterranean*, *Dallas*, *Panama*, *Sassari*, *Cagliari*, *and Birmingham*	Toxicity	3
Daptomycin	*ABCB1*	*rs1045642*	Metabolism/PK	3
Minocycline	*HLA-B*	HLA-B*35:02	Toxicity	3
Metronidazole	*CYP2A6*	CYP2A6*1, CYP2A6*2, CYP2A6*9, and CYP2A6*17	Metabolism/PK	3
Chloramphenicol	*G6PD*	*G6PD A- 202A_376G, G6PD B (reference)*	Toxicity	3
	*MT-RNR1*	*rs28358569* and *rs1556422499*	Toxicity	3
	*GSTT1*	*GSTT1 non-null* and *GSTT1 null*	Toxicity	4
Penicillin G	*HLA-B*	HLA-B*55:01	Toxicity	3
Penicillin V	*HLA-B*	HLA-B*55:02	Toxicity	3
Flucloxacillin	*HLA-B*	HLA-B*57:01	Toxicity	1A
	*NR1I2*	*rs3814055*	Toxicity	3
Dicloxacillin	*ABCB1*	*rs2032582* and *rs1045642*	Metabolism/PK and others	3
Clindamycin	*HLA-B*	HLA-B*51:01, HLA-B*15:27	Toxicity	3
Vancomycin	*HLA-A*	HLA-A*32:01	Toxicity	3
Geldanamycin	*EGFR*	*rs712829*	Efficacy	3

Here, evidence level 1A-(High), Level 3-(low) and level 4-(Unsupported); PK-Pharmacokinetics.

**TABLE 3:** Current PGx-based clinical annotations of various antibiotic–gene pairs with the PharmGKB level of evidence.

Drug	Gene	Variants	Clinical annotation	Level of evidence
Rifampicin	*GSTT1*	*GSTT1 non-null* and *GSTT1 null*	Toxicity	4
	*TNF*	*rs1800629*	Toxicity	3
	*SLCO1B1*	*rs11045819*, *rs2306283*, *rs4149032*, *rs4149056*, SLCO1B1*1, and SLCO1B1*15	Metabolism/PK and toxicity	3
	*RIPOR2*	*rs10946737* and *rs10946739*	Toxicity	3
	*NR1I2*	*rs2472677*	Other	3
	*NOS2*	*rs11080344*	Toxicity	3
	*NAT2*	*rs4646244*, *rs1041983*, and *rs1041983*	Metabolism/PK and toxicity	3
	*GSTP1*	*rs1695*	Toxicity	3
	*CYP2C9*	*rs9332096*	Toxicity	3
	*CYP2C19*	*rs4986893*	Toxicity	3
	*CYP2B6*	CYP2B6*1 and CYP2B6*6	Toxicity	3
	*CUX2*	*rs7958375*	Toxicity	3
	*AGBL4*	*rs320003*, *rs393994*, *and rs319952*	Toxicity	3
	*AADAC*	*rs1803155*	Metabolism/PK	3
Pyrazinamide	*CYP2B6*	CYP2B6*1 and CYP2B6*6	Toxicity	3
	*CYP2C19*	*rs4986893*	Toxicity	3
	*CYP2C9*	*rs9332096*	Toxicity	3
	*NAT2*	*rs4646244*, *rs1041983*, and *rs1041983*	Metabolism/PK and toxicity	3
	*TNF*	*rs1800629*	Toxicity	3
	*GSTT1*	*GSTT1 non-null* and *GSTT1 null*	Toxicity	4
Isoniazid	*NAT2*	NAT2*4, NAT2*5, NAT2*6, NAT2*7, NAT2*14, and NAT2*16	Toxicity	1B
	*NAT2*	NAT2*4, NAT2*5, NAT2*6, NAT2*7, NAT2*14, NAT2*16, and NAT2*39	Metabolism/PK	2A
	*ABCB1*	*rs1045642*	Toxicity	3
	*BACH1*	*rs2070401*	Toxicity	3
	*CYP2B6*	CYP2B6*1 and CYP2B6*6	Toxicity	3
	*CYP2C19*	*rs4986893*	Toxicity	3
	*CYP2C9*	*rs9332096*	Toxicity	3
	*GSTP1*	*rs1695*	Toxicity	3
	*MAFK*	*rs4720833*	Toxicity	3
	*NAT2*	*rs1041983*, *rs4646244*, *rs1799930*, *rs1208*, *rs1801280*, *rs1799931*, and *rs1799929*	Metabolism/PK and toxicity	3
	*NOS2*	*rs11080344*	Toxicity	3
	*TNF*	*rs1800629*	Toxicity	3
	*XPO1*	*rs11125883*	Toxicity	3
	*GSTT1*	*GSTT1 non-null and GSTT1 null*	Toxicity	4
Ethambutol	*CYP2B6*	CYP2B6*1 and CYP2B6*6	Toxicity	3
	*CYP2C19*	*rs4986893*	Toxicity	3
	*CYP2C9*	*rs9332096*	Toxicity	3
	*NAT2*	*rs4646244* and *rs1041983*	Metabolism/PK and toxicity	3
	*TNF*	*rs1800629*	Toxicity	3
	*GSTT1*	*GSTT1 non-null* and *GSTT1 null*	Toxicity	4
Dapsone	*HLA-B*	HLA-B*13:01	Toxicity	2A
	*HLA-A*	HLA-A*24:02	Toxicity	3
	*HLA-B*	HLA-B*15:02	Toxicity	3
	*HLA-DRB1*	*rs17211071*, *rs701829*, *rs201929247*, HLA-DRB1*15:01, and HLA-DRB1*16:02	Toxicity	3
	*G6PD*	*rs1050828*	Toxicity	4
Co-trimoxazole	*HLA-B*	HLA-B*13:01, HLA-B*15:02, and HLA-B*38:02	Toxicity	2A
	*HLA-C*	HLA-C*06:02, HLA-C*07:27, and HLA-C*08:01	Toxicity	2B
	*GSTM1*	*GSTM1 non-null* and *GSTM1 null*	Toxicity	3
	*HLA-B*	HLA-B*07:02	Toxicity	4
	*HLA-C*	HLA-C*07:02	Toxicity	3
	*NAT2*	NAT2*4, NAT2*5, NAT2*6, NAT2*7, NAT2*14, NAT2*16, *rs1799930*, *and rs1799931*	Toxicity	3
	*G6PD*	*G6PD B (reference)*, *G6PD Canton*, *Taiwan-Hakka*, *Gifu-like*, *and Agrigento-like*	Toxicity	4
Sulfasalazine	*ABCG2*	*rs2231142* and *rs72552713*	Metabolism/PK and efficacy	3
	*G6PD*	*G6PD A- 202A_376G* and *G6PD B (reference)*	Toxicity	3
	*HLA-B*	HLA-B*39:01, HLA-B*13:01, and HLA-B*15:05	Toxicity	3
	*MTR*	*rs1805087*	Efficacy	3
Daunorubicin	*SLC28A3*	*rs7853758*	Toxicity	2B
	*ABCB1*	*rs2032582*	Efficacy	3
	*BMP7*	*rs79085477*	Toxicity	3
	*DOK5*	*rs117532069*	Toxicity	3
	*DROSHA*	*rs639174*	Toxicity	3
	*GATA3*	*rs3824662*	Toxicity	3
	*LINC00251*	*rs141059755*	Toxicity	3
	*RARG*	*rs2229774*	Toxicity	3
	*SLCO1B1*	*rs2291075*	Efficacy	3
	*NOS3*	*rs1799983*	Efficacy	3
	*NRP2*	*rs10932125*	Other	3
Doxorubicin	*SLC28A3*	*rs7853758*	Toxicity	2B
	*ABCB1*	*rs2229109*, *rs1045642*, *rs2032582*, *rs1128503*, *rs4148737*, and *rs45511401*	Efficacy and toxicity	3
	*ABCC2*	*rs8187710*, *rs3740066*, *rs17222723*, *rs2273697*, and *rs717620*	Toxicity and efficacy	3
	*ABCC3*	*rs4148416*	Efficacy	3
	*ABCC4*	*rs9561778*	Toxicity	3
	*ABCG2*	*rs2231142*	Toxicity	3
	*AKR1C3*	*rs1937840*	Efficacy	3
	*ALDH1A1*	*rs6151031*	Efficacy	3
	*ALDH3A1*	*rs2228100*	Toxicity	3
	*ATM*	*rs1801516*	Toxicity	3
	*BMP7*	*rs79085477*	Toxicity	3
	*CBR1*	*rs9024* and *rs20572*	Dosage, toxicity, and metabolism/PK	3
	*CBR3*	*rs8133052*	Toxicity and efficacy	3
	*CCND1*	*rs9344*	Efficacy	3
	*CLCN6* and *MTHFR*	*rs1801133*	Toxicity	3
	*CYBA*	*rs4673*	Toxicity and efficacy	3
	*CYP1B1*	*rs1056836*	Toxicity	3
	*CYP2B6*	*rs3745274*, *rs12721655*, *and rs3211371*	Dosage, efficacy, and toxicity	3
	*CYP2C19*	*rs4244285* and *rs12248560*	Toxicity and Efficacy	3
	*DOK5*	*rs117532069*	Toxicity	3
	*ERCC1*	*rs11615* and *rs3212986*	Toxicity	3
	*ERCC2*	*rs13181*	Toxicity	3
	*GATA3*	*rs3824662*	Efficacy	3
	*GSTA1*	*rs3957357*	Efficacy	3
	*GSTM1*	*GSTM1 non-null* and *GSTM1 null*	Toxicity and efficacy	3
	*GSTP1*	*rs1695*	Toxicity and efficacy	3
	*GSTT1*	*GSTT1 non-null* and *GSTT1 null*	Efficacy	3
	*LINC00251*	*rs141059755*	Toxicity	3
	*MTHFD1*	*rs2236225*	Efficacy	3
	*NCF4*	*rs1883112*	Toxicity	3
	*NOS3*	*rs1799983* and *rs2070744*	Efficacy	3
	*NQO2*	*rs1143684*	Efficacy	3
	*RAC2*	*rs13058338*	Toxicity	3
	*RARG*	*rs2229774*	Toxicity	3
	*SLC22A16*	*rs714368*, *rs6907567*, *rs12210538*, and *rs723685*	Toxicity, dosage, and efficacy	3
	*SLCO1B1*	*rs4149056*	Toxicity	3
	*TMEM43* and *XPC*	*rs2228001*	Toxicity	3
	*XRCC1*	*rs25487*	Toxicity	3
Epirubicin	*CBR3*	*rs112783657* and *rs74743371*	Toxicity	3
	*CCNK*	*rs77769901*	Toxicity	3
	*CYP1B1*	*rs1056836*	Toxicity and efficacy	3
	*CYP2C8*	*rs117458836*	Toxicity	3
	*FOXO1*	*rs144991623*	Toxicity	3
	*GNL3*	*rs112242273*	Toxicity	3
	*GSTP1*	*rs1695*	Toxicity and efficacy	3
	*HMMR*	*rs299313*, *rs299314*, and *rs299293*	Toxicity	3
	*INSR*	*rs142244113* and *rs41412545*	Toxicity	3
	*IRS1*	*rs115457081*	Toxicity	3
	*MDM4*	*rs1563828*	Efficacy	3
	*NOS1*	*rs149212925*	Toxicity	3
	*NOS3*	*rs1799983*	Efficacy	3
	*NQO1*	*rs1800566*	Efficacy	3
	*PERP*	*rs78428806*, *rs117101815*, *rs9402944*, and *rs9389568*	Toxicity	3
	*PIGB*	*rs12050587*	Toxicity	3
	*PIK3R2*	*rs117951771*, *rs148235907*, *rs138602176*, *rs150688309*, *rs79430272*, *rs55633228*, *rs118129530*, *rs56022120*, *rs117341846*, *rs148013902*, *rs145623321*, *rs58695150*, and *rs8110364*	Toxicity	3
	*PON1*	*rs662*	Efficacy	3
	*PPP2R5D*	*rs3805945*	Toxicity	3
	*RBX1*	*rs141084494*	Toxicity	3
	*SLCO1B1*	*rs4149056*	Toxicity	3
	*TOP2A*	*rs181501757*	Toxicity	3
	*TP53*	*rs4968187*	Toxicity	3
Mitoxantrone	*TP53AIP1*	*rs118088833*	Toxicity	3
	*GALNT14*	*rs9679162* and *rs12613732*	Efficacy	3
	*SLCO1B1*	*rs2291075*	Efficacy	3
Bleomycin	*ABCB1*	*rs1045642* and *rs2229109*	Toxicity	3
	*BLMH*	*rs1050565*	Toxicity	3
	*CYP3A4*	*rs2740574*	Toxicity	3
	*ERCC1*	*rs3212986* and *rs11615*	Toxicity	3
	*ERCC2*	*rs1799793*, *rs238406*, and *rs13181*	Toxicity	3
	*GSTM1*	*GSTM1 non-null* and *GSTM1 null*	Toxicity and efficacy	3
	*GSTP1*	*rs1695*	Toxicity	3

PGx, pharmacogenomics; PK, pharmacokinetics.

Although most of the annotations were assigned evidence level-3 (low), for a few antibiotics, we also identified some moderate (2A and 2B) and high (1A and 1B) levels of evidence. Aminoglycosides (amikacin, neomycin, gentamicin, kanamycin, streptomycin, and tobramycin) had a level-1A association for toxicity (ototoxicity) with different variants of *MT-RNR1*—*rs267606617* being the variant common to all of them. Other variants are outlined in Tables 2, 3. For flucloxacillin, we observed another level-1A association with HLA-B*57:01 for drug-induced liver injury. For isoniazid induced toxicity, level-1B evidence was assigned with the *NAT2* for the variants NAT2*1, NAT2*4, NAT2*5, NAT2*6, NAT2*7, NAT2*14, and NAT2*16.

Similarly, level-2A evidence was assigned with isoniazid for metabolism/PK for various variants of the *NAT2* gene (i.e., NAT2*1, NAT2*4, NAT2*5, NAT2*6, NAT2*7, NAT2*14, NAT2*16, and NAT2*39). For drug-induced toxicity, an evidence level of 2A was assigned with various variants of *HLA-B* for co-trimoxazole (HLA-B*13:01, HLA-B*15:02, and HLA-B*38:02) and dapsone (HLA-B*13:01). Co-trimoxazole also had a level-2B association for toxicity with HLA-C*06:02, HLA-C*07:27, and HLA-C*08:01. Anthracycline antibiotics (doxorubicin and daunorubicin) had a level-2A association for drug-induced toxicity with *SLC28A3* (*rs7853758*).

**FIGURE 2:**
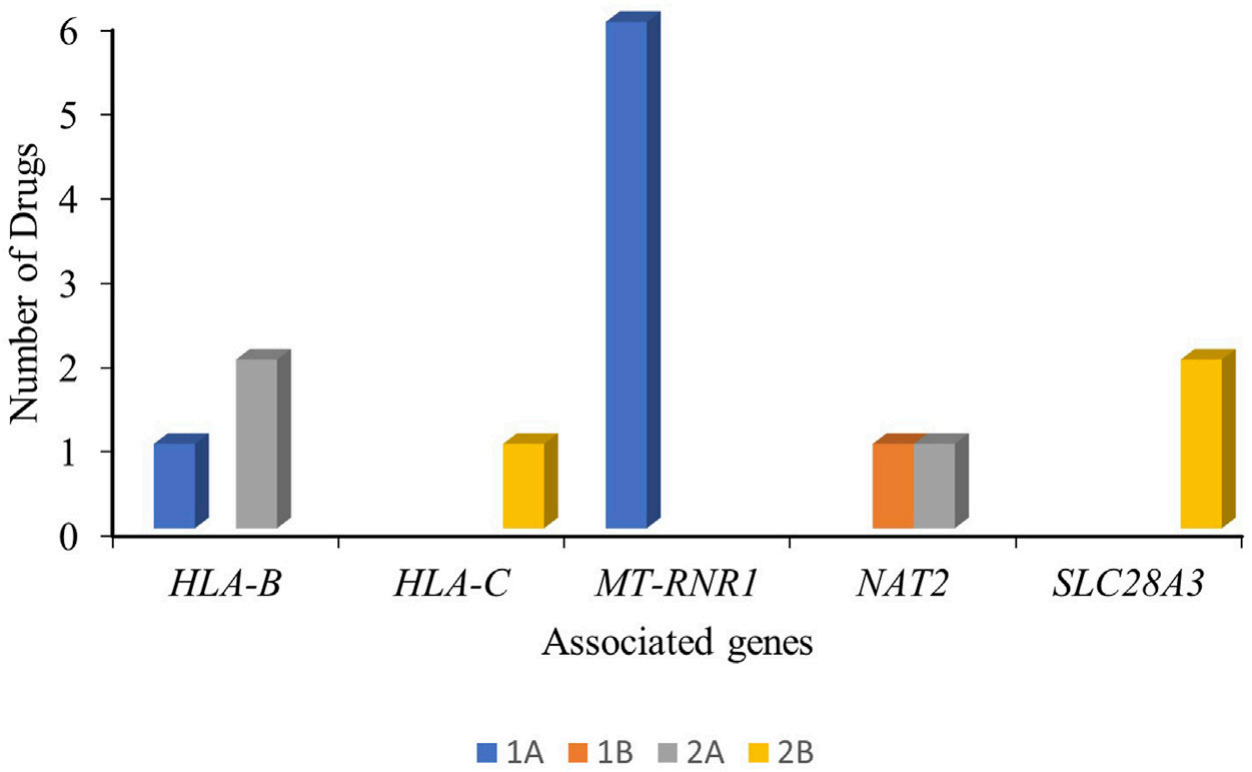
Clinical annotations (level-1 and level-2) of the antibiotic drugs and the associated genes with their PharmGKB evidence level.

Considering the overall clinical annotations for antibiotics, we identified *HLA-B* (one level-1A, two level-2A, and eight level-3 associations), *MT-RNR1* (six level-1A and two level-3 associations), and *NAT2* (one level-1B, one level-2B, and five level-3 associations) as concerning genes for the safety and effectiveness of the antibiotic drug. The clinical annotations of level-1 and level-2 for antibiotics are outlined in Figure 2.

**FIGURE 3:**
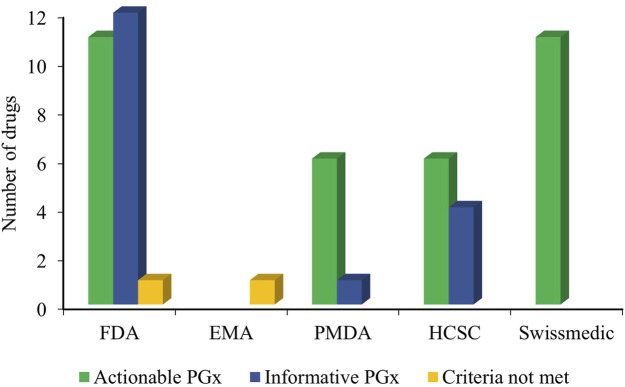
Overall PGx-based drug label of the antibiotics from the FDA, EMA, PMDA, HCSC, and Swissmedic (HCSC, Health Canada Santé Canada; FDA, US Food and Drug Administration; Swissmedic, Swiss Agency of Therapeutic Products; PMDA, Pharmaceuticals and Medical Devices Agency, Japan; EMA, European Medicines Agency; PGx, Pharmacogenomics).

The PharmGKB curates and presents the PGx-based drug labels on its site. These labels are sourced from the FDA, EMA, PMDA, HCSC, and Swissmedic and are presented as testing required, testing recommended, actionable PGx, informative PGx, no clinical PGx, and criteria not met (PharmGKB, 2025b). Our search across the PharmGKB website revealed PGx label information for at least 27 antibiotic drugs, considering the polymorphisms of at least 6 genes (*MT-RNR1*, *G6PD*, *NAT2*, *CYB5R3*, *CYP3A4*, and *HLA-B*) involved. These labels are presented in Table 4. Although the majority of the drugs were labeled as actionable PGx, none were labeled as no clinical PGx, testing required, or testing recommended. Actionable PGx entails contraindication, dose alteration, alternative therapy, or other management for individuals with a specific metabolizer phenotype or genotype (if known). This label, however, does not recommend phenotype or genotype testing prior to the use of the drug. The informative PGx label provides information on a particular variant/gene/phenotype/protein that can potentially affect the metabolism, concentration, and frequency of side effects or impose a general risk for the patients. However, this label provides no further guidance for the actions to be undertaken in such situations (PharmGKB). The overall statistics of the PGx label of antibiotics are shown in Figure 3. The majority of these labels are sourced from the FDA-approved drug label with at least 11 actionable PGx and 12 informative PGx for antibiotic drugs. Swissmedic, with at least 11 actionable PGx, is another important source for PGx-based drug labels for antibiotics.

**TABLE 4:** PGx drug label information for antibiotics.

Drug	Gene	PGx label information	Recommending body
Amikacin	*MT-RNR1*	Actionable PGx	FDA
Ciprofloxacin	*G6PD*	Actionable PGx	Swissmedic
Co-trimoxazole	*G6PD*	Actionable PGx	PMDA and Swissmedic
		Informative PGx	FDA and HCSC
	*NAT2*	Informative PGx	FDA
Dapsone	*CYB5R3*	Actionable PGx	FDA and HCSC
	*G6PD*	Actionable PGx	FDA, PMDA, and HCSC
Erythromycin	*G6PD*	Informative PGx	FDA
Flucloxacillin	*HLA-B*	Actionable PGx	Swissmedic
Gentamicin	*MT-RNR1*	Actionable PGx	FDA
Isoniazid	*NAT2*	Informative PGx	FDA and PMDA
Levofloxacin	*G6PD*	Actionable PGx	Swissmedic
Mafenide	*G6PD*	Informative PGx	FDA
Moxifloxacin	*G6PD*	Actionable PGx	Swissmedic
Nalidixic acid	*G6PD*	Actionable PGx	FDA and PMDA
Neomycin	*MT-RNR1*	Actionable PGx	FDA
Nitrofurantoin	*G6PD*	Actionable PGx	FDA, HCSC, and Swissmedic
Norfloxacin	*G6PD*	Actionable PGx	Swissmedic
		Informative PGx	FDA and HCSC
Ofloxacin	*G6PD*	Actionable PGx	Swissmedic
Plazomicin	*MT-RNR1*	Actionable PGx	FDA
Pyrazinamide	*NAT2*	Informative PGx	FDA
Rifampicin	*NAT2*	Informative PGx	FDA
Streptomycin	*MT-RNR1*	Actionable PGx	FDA
Sulfadiazine	*G6PD*	Actionable PGx	HCSC, PMDA, and Swissmedic
		Informative PGx	FDA
Sulfasalazine	*G6PD*	Actionable PGx	FDA, PMDA, HCSC, and Swissmedic
	*NAT2*	Informative PGx	FDA and HCSC
Sulfisoxazole	*G6PD*	Informative PGx	FDA
Tobramycin	*MT-RNR1*	Actionable PGx	FDA and HCSC
Trimethoprim	*G6PD*	Actionable PGx	PMDA and Swissmedic
		Informative PGx	HCSC
	*G6PD* and *NAT2*	Informative PGx	FDA
Ceftriaxone	*CYB5R3* and *G6PD*	Criteria not met	FDA
Telithromycin	*CYP3A4*	Criteria not met	EMA

HCSC, Health Canada Santé Canada; FDA, US Food and Drug Administration; Swissmedic, Swiss Agency of Therapeutic Products; PMDA, Pharmaceuticals and Medical Devices Agency, Japan; EMA, European Medicines Agency; PGx, Pharmacogenomics.

### 3.4 Current state of PGx-based therapeutic and testing guidelines for antibiotics

The search for PGx-based guidelines across CPIC, DPWG, and CPNDS revealed at least six genes i.e., *HLA-B*, *MT-RNR1*, *G6PD*, *RARG*, *SLC28A3*, and *UGT1A6*. These PGx working bodies recommend therapy or testing for optimizing the effectiveness of several antibiotics based on the genetic variants of these six genes (Aminkeng et al., 2016; Gammal et al., 2023; Mcdermott et al., 2022, Dpwg, 2025b). For flucloxacillin-induced liver injury, DPWG deemed genotyping for HLA-B*57:*01* to be beneficial and recommended alternative medicine for HLA-B*57:01-positive patients when bilirubin and/or liver enzyme levels are found elevated (Dpwg, 2025b). For aminoglycoside-induced hearing loss, CPIC provided a guideline considering the genotype of *MT-RNR1*, where they classified people into the categories normal, increased, and uncertain risk of aminoglycoside-induced hearing loss based on their genotype. In patients at increased risk, aminoglycoside use is strongly discouraged unless both the lack of safer alternatives and the severity of the infection outweigh the risk of ototoxicity (Mcdermott et al., 2022).

Based on the polymorphism in *G6PD*, the CPIC provided therapeutic guidelines for dapsone and nitrofurantoin. They classified individuals into normal, deficient, and deficient in chronic non-spherocytic hemolytic anemia (CNSHA) groups and variable and indeterminate groups based on the genotypes of *G6PD.* Avoidance of dapsone use is strongly recommended in deficient and deficient in CNSHA groups. On the contrary, for those deficient in the CNSHA group, avoidance of nitrofurantoin use is moderately recommended. They also suggested that in the deficient group, nitrofurantoin can be used in a standard dose, optionally with close monitoring for anemia (Gammal et al., 2023).

CPNDS, on the other hand, provided a guideline for anthracycline (doxorubicin, daunorubicin, and others)-induced cardiotoxicity based on the polymorphism of *RARG*, *SLC28A3*, *and UGT1A6.* They classified individuals according to their genotype into low, moderate, and high-risk groups. For the high-risk group, comprising individuals carrying *RARG rs2229774A* or UGT1A6*4, the CPNDS strongly recommended increased monitoring frequency and appropriate management of associated cardiovascular risk factors. They moderately encouraged the use of dexrazoxane and liposome-enclosed anthracycline preparations. As optional recommendations, they suggest slower infusion rates or continuous infusion, use of cardioprotective agents, or choosing alternative therapy with comparable efficacy (if available). For children receiving doxorubicin or daunorubicin therapy, CPNDS moderately recommended genetic testing for *RARG rs2229774A*, *SLC28A3 rs7853758*, and UGT1A6*4 rs17863783 variants. They, however, did not recommend genetic testing for children and adults receiving other types of anthracyclines (Aminkeng et al., 2016).

More details on these guidelines provided by DPWG, CPIC, and CPNDS are presented in Table 5.

**TABLE 5:** Current PGx-based therapeutic and testing guidelines for antibiotics provided by the CPIC, CPNDS, and DPWG.

Drug	Gene	Likely phenotype	Genotype	Recommending body	Therapeutic and dosing recommendation	Classification of recommendations	Testing recommendation	Reference
Flucloxacillin	*HLA-B*	Positive/negative	HLA-B*5701	DPWG	HLA-B*5701-positive patients have an 80-fold higher risk of flucloxacillin-induced liver injury It is recommended to monitor patient’s liver function regularly and opt for an alternative if the liver enzymes and/or bilirubin levels are increased	-	It is recommended to consider genotyping these patients before (or directly after) drug therapy has been initiated to guide drug selection	https://www.pharmgkb.org/guidelineAnnotation/PA166182810
Amikacin, dibekacin, gentamicin, kanamycin, neomycin, netilmicin, paromomycin, plazomicin, ribostamycin, streptomycin, and tobramycin	*MT-RNR1*	Increased risk of aminoglycoside-induced hearing loss	*m.1095T>C m.1494C>T m.1555A>G*	CPIC	Avoid using aminoglycoside antibiotics except where the severity of infection and unavailability of effective or safe alternative therapies outride the significant risk of permanent hearing loss	Strong	-	2022 McDmmm
		Normal risk of aminoglycoside-induced hearing loss	*m.827A>G*		It is advised to use aminoglycoside antibiotics at standard doses for the shortest possible course with careful therapeutic dose monitoring. Hearing loss should be regularly evaluated following the local guidance	Strong		
Dapsone	*G6PD*	Normal	An individual having one X chromosome carrying a non-deficient allele; an individual having two non-deficient alleles	CPIC	Based on the *G6PD* status, dapsone needs not to be avoided	Strong	-	2022 Gammal
		Deficient	An individual having one X chromosome carrying a deficient allele. An individual inheriting two deficient alleles or one class I allele and one class II or III allele		Avoidance of dapsone is recommended	Strong		
		Deficient with CNSHA	An individual having one X chromosome carrying a deficient allele; an individual inheriting two deficient alleles		Avoidance of dapsone is recommended	Strong		
		Variable	An individual inheriting one non-deficient allele and one deficient allele		Measuring the enzyme activity is necessary for ascertaining the *G6PD* status, and the use of drug should be according to the recommendations on the basis of the activity-based phenotype	Moderate		
		Indeterminate	An individual having at least one uncertain function allele		Measuring the enzyme activity is necessary for ascertaining the G6PD status, and the use of drug should be according to the recommendations on the basis of the activity-based phenotype	Moderate		
Nitrofurantoin	*G6PD*	Normal	An individual having one X chromosome carrying a non-deficient allele. An individual having two non-deficient alleles	CPIC	Based on the *G6PD* status, nitrofurantoin need not to be avoided	Strong	-	2022 Gammal
		Deficient	An individual having one X chromosome carrying a deficient allele; an individual inheriting two deficient alleles or one class I allele and one class II or III allele		Nitrofurantoin is recommended at standard doses, with close monitoring for anemia	Optional		
		Deficient with CNSHA	An individual having one X chromosome carrying a deficient allele; an individual inheriting two deficient alleles		Avoidance of nitrofurantoin is advised	Moderate		
		Variable	An individual inheriting one non-deficient (class IV) allele and one deficient (class I– III) allele (B/Bangkok, B/Mediterranean, B/A, IV/I, IV/II, and IV/III)		Measuring the enzyme activity is necessary for ascertaining the G6PD status, and the use of drug should be according to the recommendations on the basis of the activity-based phenotype	Moderate		
		Indeterminate	An individual having at least one uncertain function allele		Measuring the enzyme activity is necessary for ascertaining the G6PD status, and the use of drug should be according to the recommendations on the basis of the activity-based phenotype	Moderate		
Anthracycline (doxorubicin, daunorubicin, and others)	*RARG*, *SLC28A3*, and *UGT1A6*	High risk	*RARG rs2229774A* and UGT1A6*4	CPNDS	Increasing the monitoring frequency is advised. Vigorous monitoring and proper management of the cardiovascular risk factors (e.g., diabetes, obesity, arterial hypertension, lipid disorders, coronary artery disease, and peripheral vascular disease) are recommended	Level A (strong)	Genetic testing for *RARG rs2229774*, *SLC28A3 rs7853758*, and UGT1A6*4 rs17863783 variants is recommended in children being treated with doxorubicin or daunorubicin (level B, moderate). In children and adults receiving other types of anthracyclines, genotyping is not currently recommended (level C, optional)	2016 - Aminkeng
					Dexrazoxane should be prescribed. Use of anthracycline preparations encapsulated in liposome can be considered	Level B (moderate)		
					Continuous infusions or slower rates of infusion must be included. The use of cardiotoxic types of anthracyclines should be reduced. Use of other cardioprotective agents can be considered. Alternative chemotherapy regimens can be prescribed for particular type of tumors, where these alternative regimes exhibited comparable efficacy	Level C (optional)		
		Low risk	*SLC28A3 rs7853758A*		A normal follow-up is advised	Level A (strong)		
		Moderate risk	All other patients		Increase the frequency of monitoring	Level A (strong)		

CPIC, Clinical Pharmacogenetics Implementation Consortium; DPWG, Dutch Pharmacogenetics Working Group; CPNDS, Canadian Pharmacogenomics Network for Drug Safety.

## 4 Discussion

This study identified a total of 65 clinical studies evaluating the genetic impact in producing different drug-induced adverse effects associated with antibiotic drugs. These studies provide a wide range of evidence reinforcing the need for PGx-based antibiotic therapy in clinical practice to achieve precision medicine. This evidence base explored a variety of gene variants associated with the ADRs—for example, beta-lactam-induced hypersensitivity reaction (with a varying OR of 1.36–5.1), flucloxacillin-induced DILI (associated with several *HLA* genes with ORs ranging from 1.86 to 79.21), anti-tuberculosis drug-induced hepatotoxicity (OR range 0.10–9.57), anthracycline-induced cardiotoxicity (reporting a varied ORs from 0.14 to 7.98), co-trimoxazole-induced SCARs (for a limited number of *HLA* genes with an OR range of 4.05–43.57), etc. A few of the protective biomarkers were identified during the literature search, such as NAT2*5 and *NAT2 (rs1495741)* (for isoniazid-induced liver injury, OR = 0.69 and 0.10, respectively), *SLC22A16 T>C (rs714368)* for doxorubicin-induced neutropenic and leukopenia (OR = 0.31 and 0.18, respectively), *NQO1609TT* (for epirubicin-induced anemia OR = 0.34 and grade 2–4 toxicity OR = 0.33), *SLC28A3 (rs7853758)*, *SLC28A3 (rs885004)*, *ABCC10 (rs1214763)*, *CYP2J2 (rs2294950)*, *FMO2 (rs2020870)*, *GPX3 (rs2233302)*, *GSTM3 (rs12059276)*, *SLC28A3 (rs7853758)*, *SLC10A2 (rs9514091)*, *SLC28A3 (rs4877847)*, *SLC22A17 (rs4982753)*, *SLC22A7 (rs4149178)*, *SOD2 (rs7754103)*, *SPG7 (rs2019604)*, and *XDH (rs4407290)* (for anthracycline-induced cardiotoxicity, OR = 0.46, 0.42, 0.34, 0.41, 0.14, 0.27, 0.37, 0.31, 0.43, 0.60, 0.52, 0.41, 0.30, 0.39, and 0.26, respectively) (Chan et al., 2017; Chaturvedi et al., 2015; Ebaid et al., 2024; Nicoletti et al., 2021b; Visscher et al., 2015; Visscher et al., 2012). We also explored the PharmGKB evidence level and PGx label information, which provided similar information on the genetic associations for the antibiotic drug-induced ADRs. However, to date, the clinical and dosing guidelines have been suggested for only a limited number of antibiotic drugs, with the aim of optimizing safety and effectiveness while reducing the incidence of ADRs through prediction. The findings of the current study, therefore, encourage policymakers to consider the growing evidence and take the necessary measures for its clinical adoption.

Although some robust literature-based associations were identified in the included studies, most of them provided preliminary associations of the genetic variants and adverse effects and recommended further exploration with a large number of subjects across the population for a comprehensive understanding, validation, and translation into implementable clinical guidelines (Amorim et al., 2023; An et al., 2012; Calcagno et al., 2019; Goldman et al., 2022; Guéant-Rodriguez et al., 2008; Gupta et al., 2013; Krebs et al., 2020; Nicoletti et al., 2021a; Nyangwara et al., 2024; Park et al., 2024; Sukasem et al., 2020; Suvichapanich et al., 2019; Tempark et al., 2017; Thomas et al., 2025; Vuilleumier et al., 2006; Wang et al., 2024b; Yang et al., 2017; Yuliwulandari et al., 2016). However, such proper large-scale follow-up studies were scarce, keeping these reported preliminary associations largely unexplored, which may contribute to the limited number of clinical guidelines available. Nevertheless, there are several antibiotic candidates with various genetic associations replicated in multiple studies and have moderate to high (level-1 and level-2) PharmGKB evidence level and PGx drug label information. For example, the association between isoniazid and the *NAT2* genetic polymorphism has been well studied for toxicity, carries a high PharmGKB evidence level-1B, and has been labeled with informative PGx by the FDA and PMDA (Ben Fredj et al., 2017; Chan et al., 2017; Kasamatsu et al., 2025; Nicoletti et al., 2021b; Thomas et al., 2025). Similarly, the association between co-trimoxazole and *HLA* genes for SCARs has been reported in multiple clinical studies and has a moderate PharmGKB evidence level of 2A (for *HLA-B*) and 2B (for *HLA-C*) for drug-induced toxicity. However, this genetic association with *HLA* has no PGx label information (Goldman et al., 2022; Sukasem et al., 2020). It is evident that even after having some considerable and growing evidence for certain genetic associations for antibiotics and toxicity, sufficient measures are not being undertaken to translate them into clinical use. It is about time for the international PGx working bodies to develop PGx-dosing guidelines so that clinicians can easily incorporate recommendations into routine clinical practice.

As of now, no antibiotic drug has a testing-required or recommended label by the FDA, EMA, PMDA, HCSC, or Swissmedic. Nevertheless, several studies reported the importance of genetic testing in the prediction and management of adverse effects associated with antibiotics. For example, Gupta et al. (2013) informed that the early detection of *GSTM1 and T1 null* may help lower ATD-induced hepatotoxicity. To reduce the risk of AT-DILI, Yuliwulandari et al. (2016) recommended the *NAT2* genotype and corresponding phenotype determination. For customizing the anthracycline therapy in cancer, Ebaid et al. (2024) emphasized the importance of genetic testing for *SLC22A16* and *CBR1*. A prediction model based on both genetic and clinical risk factors was deemed beneficial by Visscher et al. (2013) in anthracycline therapy for identifying risk profiles for cardiotoxicity. For vancomycin-induced DRESS, Konvinse et al. (2019) stated that HLA-A*32:01 testing may improve safety and efficacy. For levofloxacin-induced SCARs, Jiang et al. (2023) informed prospective screening of *serotype B13*, and prescribing alternative drug therapy for the carriers significantly reduces the incidence of adverse effects. Satapornpong et al. (2021) supported the genotyping of the HLA-B*13:01 allele to avoid SCARs with dapsone therapy in the Asian population. Asif et al. (2024) recommended considering the screening of HLA-A*32:01 for risk stratification in long-term therapy with vancomycin (Asif et al., 2024; Blanco et al., 2012; Ebaid et al., 2024; Göpel et al., 2014; Gupta et al., 2013; Jiang et al., 2023; Konvinse et al., 2019; Satapornpong et al., 2021; Schiuma et al., 2025; Visscher et al., 2013; Wang et al., 2024b; Yuliwulandari et al., 2016).

Another limiting factor for the adoption of PGx in clinical practice for antibiotic therapy is the paucity of cost-effectiveness studies. Health economics plays a vital role in supporting policymakers in allocating limited resources, and therefore, cost-effectiveness studies are essential for evidence-based decision-making (Kategeaw et al., 2023; Leelahavarong et al., 2019). One such cost-effectiveness analysis conducted by Kategeaw et al. (2023), for preventing SCARs with co-trimoxazole therapy in HIV-infected Thai patients, revealed that the screening of HLA-B*13:01 before initiating the therapy was not likely to be cost-effective. Similar cost-effectiveness studies for the important antibiotic-genetic variant pairs in diverse populations are warranted to provide a comprehensive overview of the effects of PGx in antibiotic therapy and subsequent adoption in clinical practice.

Several complex traits, such as the sensitivity to adverse reactions and efficacy of the drug, are sometimes attributable to several different genetic variants. Owing to the remarkable progress in genome sequencing and genome-wide association studies, several polygenic risk scores, including some related to PGx, have been developed (Cross et al., 2022; Evans et al., 2009). For antibiotics, such multigene effects have also been recorded. For example, *GSTM1* and *T1 null* genotypes had a significant association with ATD-induced hepatotoxicity (OR = 7.18, 95% CI: 1.7–32.6, *p* = 0.007), and for isoniazid-induced hepatotoxicity, individuals with both *NAT2* slow acetylator and *CYP2E1 DraI C/D* had an elevated risk (Ben Fredj et al., 2017; Gupta et al., 2013). Exploring these and other genetic associations for different antibiotic drugs and further developing polygenic risk scores for them can be a rational approach for adopting PGx-based antibiotic use in clinical practice.

To the best of our knowledge, this is the first comprehensive review showing the current evidence of antibiotic-induced hypersensitivity reactions involving PGx. Furthermore, this review summarized the current state of PGx-based therapeutic and testing guidelines for antibiotics in clinical practice, taking into account PGx-based clinical annotations and drug label information.

Although this comprehensive review has insightful information regarding PGx associations of antibiotic-induced hypersensitivity reactions, there is a limitation of this review. The search for relevant literature was carried out in PubMed only, which may limit the possibility of obtaining all potential evidence.

## 5 Conclusion

In conclusion, this study identified at least 12 antibiotic–gene pairs (amikacin–*MT-RNR1*, gentamicin–*MT-RNR1*, kanamycin–*MT-RNR1*, streptomycin–*MT-RNR1*, neomycin–*MT-RNR1*, tobramycin–*MT-RNR1*, isoniazid–*NAT2*, dapsone–*HLA-B*, co-trimoxazole–*HLA-B* and *HLA-C*, flucloxacillin–*HLA-B*, daunorubicin–*SLC28A3*, and doxorubicin–*SLC28A3*) with moderate-to-high PharmGKB evidence level for toxicity. However, PGx-based dosing guidelines, as recommended by the CPIC, DPWG and CPNDS, are available for the following antibiotic–gene pairs*:* amikacin, gentamicin, kanamycin, streptomycin*,* neomycin, and tobramycin–*MT-RNR1*; flucloxacillin–*HLA-B*; dapsone–*G6PD*; nitrofurantoin–*G6PD*; and daunorubicin and doxorubicin–*RARG*, *SLC28A3*, and *UGT1A6*. Despite the established and growing genetic evidence for the toxicity, particularly co-trimoxazole-induced SCARs associated with *HLA-B* and *HLA-C*, dapsone-induced SCARs associated with *HLA-B*, and isoniazid-induced liver injury associated with *NAT2*, sufficient efforts have not been undertaken to translate findings into routine clinical practice. The lack of validation of preliminary genetic associations, due to the scarcity of proper follow-up and large-scale replication, represents a key setback for the PGx-based implementation of antibiotic therapy in clinical practice. More focused clinical studies, cost-effectiveness analyses, and polygenic risk score development are required for the PGx-based clinical use of antibiotics to optimize the safety and effectiveness.
